# Neuroanatomical Cerebellar Patterns in Autism Spectrum Disorder

**DOI:** 10.5152/eurasianjmed.2026.251008

**Published:** 2026-06-24

**Authors:** Aslı Beril Karakaş, Yalçın Akbulut

**Affiliations:** 1 Department of Anatomy, Kastamonu University Faculty of Medicine, Kastamonu, Türkiye; 2 Department of Anatomy, Kafkas University Faculty of Medicine, Kars, Türkiye

**Keywords:** Autism spectrum disorder, brain mapping, cerebellum, magnetic resonance imaging, sex characteristics

## Abstract

**Background:**

This study aimed to investigate cerebellar volumetric and cortical-thickness differences in adults with autism spectrum disorder (ASD) using automated segmentation techniques, with a particular focus on sex-based anatomical patterns.

**Methods:**

A total of 100 participants from the Autism Brain Imaging Data Exchange database were included, comprising 48 adults with ASD (20 females, 28 males) and 52 healthy controls (23 females, 29 males). T1-weighted structural magnetic resonance imaging scans were processed using the CERES (CEREbellum Segmentation) module of volBrain to obtain lobule-specific cerebellar volume and thickness measures. Group comparisons were performed separately for males and females using non-parametric statistical tests.

**Results:**

Adults with ASD exhibited global cerebellar volumetric enlargement alongside lobule-specific reductions and altered pseudo-cortical thickness (PCT) across multiple lobules, most prominently within anterior sensorimotor and posterior cognitive regions. Several lobules demonstrated reversed asymmetry patterns relative to controls. Sex-stratified analyses suggested differential morphometric patterns between females and males with ASD; however, these findings should be interpreted as exploratory given subgroup sample sizes.

**Conclusion:**

These findings demonstrate distinct cerebellar morphometric alterations in adults with ASD and support the utility of automated cerebellar segmentation for region-specific anatomical characterization. The results indicate group-level neuroanatomical differences involving the cerebellum, while emphasizing cautious interpretation regarding clinical or group-level distinguishing feature implications.

**Main Points**

- This study employed the CERES module to perform high-precision morphometric analysis of the cerebellum in adults with ASD.
- Significant volumetric enlargement and reversed asymmetry were detected in key lobules, particularly Lobule IX, previously implicated in autonomic and emotional processing.
- Sex-specific analyses revealed distinct cerebellar profiles in autistic males and females, suggesting potentially different patterns of cerebellar morphometry by sex; these subgroup findings should be considered exploratory given modest sample sizes and the lack of significant interaction effects after false discovery rate.
- The findings demonstrate the value of automated cerebellar segmentation in capturing subtle yet group-level neuroanatomical differences with potential translational relevance in ASD.

## Introduction

Autism spectrum disorder (ASD) is a neurodevelopmental condition marked by persistent social-communication difficulties and restricted, repetitive behaviors, with symptoms emerging early and continuing lifelong.^1^ Although multifactorial in origin, converging evidence implicates structural and functional abnormalities across several brain regions—including the cerebellum—in its pathophysiology.^2^

Traditionally viewed as a motor structure, the cerebellum is now recognized as a hub supporting cognition and affect through its extensive connections with cortical regions involved in social and executive functioning.^3^^,^^4^ Neuroimaging studies have reported cerebellar volumetric differences in ASD,^5^^,^^6^ although findings have varied, likely because of differences in imaging protocols, participant characteristics, and segmentation fidelity.^6^^-^^8^

Recent advances in automated segmentation have improved measurement precision. In particular, the CERES module of the volBrain pipeline enables reproducible quantification of total and lobular cerebellar anatomy,^9^ while large open datasets such as Autism Brain Imaging Data Exchange (ABIDE) allow broader cross-sectional characterization.^10^ Despite these methodological improvements, relatively few studies have examined cerebellar hypertrophy, asymmetry, and lobule-specific alterations within a unified, hypothesis-driven, and sex-stratified framework, leaving important gaps in how regional cerebellar features relate to the heterogeneity of ASD.

The present study therefore aimed to evaluate cerebellar morphology in adults with ASD using CERES-based volumetry from the ABIDE dataset, explicitly testing whether cerebellar enlargement, altered asymmetry, and region-specific changes represent systematic neuroanatomical signatures of ASD.

To guide the empirical focus of the study, the following a priori hypotheses were outlined:

1. Global hypothesis: Adults with ASD will show increased total cerebellar volume relative to healthy controls (HC), consistent with documented brain overgrowth.
2. Asymmetry hypothesis: Cerebellar asymmetry indices will deviate from the normative rightward dominance toward a leftward bias in ASD.
3. Regional hypothesis: Lobule-specific alterations will appear within sensorimotor (III-V), executive (VI), and autonomic–limbic (vermal IX) territories, paralleling behavioral domains affected in ASD.
4. Sex-specific hypothesis: Morphometric alterations will differ by sex, with more pronounced anterior and limbic changes in autistic females and more extensive white matter hypertrophy in autistic males.

## Material and Methods

### Participants

Adult participants were screened from the ABIDE database using predefined inclusion criteria (adult age range, availability of high-resolution T1-weighted Magnetic Resonance Imaging (MRI), and complete demographic information). In total, 135 ABIDE participants were assessed; 33 were excluded due to missing demographic/phenotypic data and/or failing the inclusion criteria, and 2 were excluded after volBrain/CERES processing because of motion artifacts, insufficient image quality, misregistration, or segmentation failure on automated and visual quality control. The final analytic sample therefore consisted of 100 adults: 48 individuals with ASD (20 females, 28 males) and 52 HC (23 females, 29 males). Importantly, sex-stratified analyses were performed within this fixed cohort without additional sex-specific exclusions. The study analyses were conducted between 01.05.2025 and 14.05.2025. Ethics approval was obtained from the Research Ethics Committee of Kafkas University (Approval No: 2025/04/09, Approval Date: 30.04.2025).

Autism spectrum disorder diagnoses were established using standardized clinical instruments [Diagnostic and Statistical Manual of Mental Disorders, Fourth Edition (DSM-IV]), Diagnostic and Statistical Manual of Mental Disorders, Fifth Edition (DSM-5), Autism Diagnostic Observation Schedule (ADOS), Autism Diagnostic Interview–Revised (ADI-R)], depending on each site's protocol. Control participants were selected on the basis of having no history of neurodevelopmental, neurological, or psychiatric disorders. Only individuals with complete demographic information and high-resolution 3T T1-weighted MRI scans were retained. Cases with motion artifacts, inadequate image quality, or segmentation failure were excluded.

Although behavioral indices such as ADOS were available for some participants, these measures were not consistently acquired across ABIDE sites. As a result, behavioral data were present for only a small subset. Conducting behavioral–morphometric correlations on this restricted subgroup would have introduced selection bias, reduced statistical power, and limited the interpretability of clinic–anatomical associations. For these reasons, such analyses were not performed, consistent with best-practice recommendations for multi-site datasets with heterogeneous phenotypic coverage.

### Magnetic Resonance Imaging Acquisition and Preprocessing

Structural MRI data were obtained from 4 ABIDE sites (Caltech, Carnegie Mellon University [CMU], HH, and GUYS), all acquired on 3T scanners using site-specific protocols. Detailed scanner parameters (repetition time [TR], echo time [TE], flip angle, voxel size) are provided in Supplementary Table 1. Although acquisition parameters varied slightly across centers, all selected scans met the minimum quality standards defined by ABIDE.

The raw NIfTI images were not subjected to local preprocessing, as volBrain requires unaltered input. Each image was processed using volBrain’s standardized pipeline, including adaptive non-local means denoizing, N4ITK bias-field correction, affine and nonlinear registration to MNI152 space, and automated skull stripping. This ensured consistent anatomical resolution and spatial normalization across subjects.

### Site Effect Harmonization (ComBat)

Because the MRI dataset originated from multiple ABIDE sites with heterogeneous scanner hardware and acquisition protocols, ComBat harmonization was applied to reduce inter-site variability while preserving biological signal. This parametric empirical Bayes method has been widely validated for multi-site neuroimaging data.^11^^,^^12^

In this study, all cerebellar morphometric metrics derived from the CERES pipeline—including regional volumes, cortical thickness, and asymmetry indices—were harmonized using ComBat. The model incorporated group (ASD vs. HC), sex, age, and total intracranial volume (TIV) as biological covariates, ensuring that variance related to diagnosis, sex, and head size was preserved. Prior to harmonization and statistical analysis, all cerebellar volumetric and PCT variables were independently verified against the original CERES output files to ensure correct variable identity and labeling. Volumetric measures derived from the CERES pipeline were expressed in cubic centimeters (cm^3^) and normalized to TIV to account for inter-individual differences in head size. PCT measures were derived from the CERES pipeline as CERES-derived regional thickness estimates and are not equivalent to histologically defined cortical thickness measures; these measures were normalized using the cubic root of TIV. Variable names, measurement units, hemispheric labels, and asymmetry definitions were cross-checked at both the raw output and statistical dataset levels. This verification step ensured a clear and unambiguous separation between volumetric and PCT metrics prior to ComBat harmonization and downstream analyses. The harmonized dataset was then used for all subsequent statistical analyses. During verification, PCT variables were confirmed to differ numerically from volume variables for the same lobule/hemisphere and that units in the exported dataset match the CERES output metadata.

### Cerebellar Segmentation, Volumetric Measurement, Cortical Thickness, and Asymmetry Analysis

**Figure 1.**
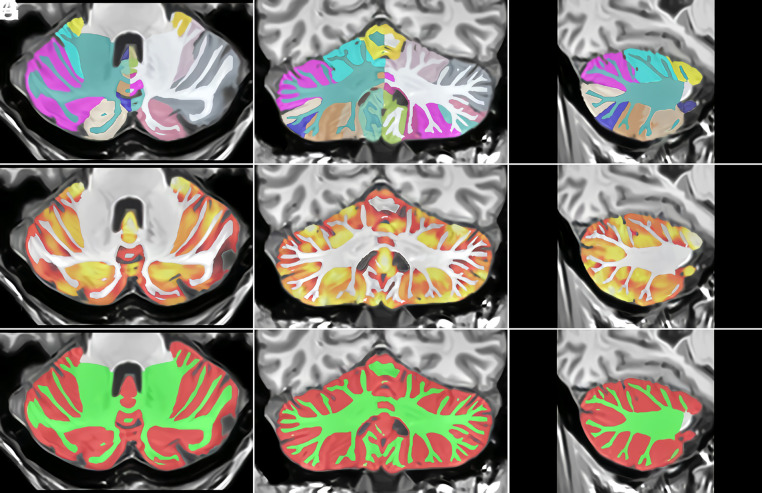
Automated cerebellar segmentation and regional parcellation using the CERES module of the volBrain platform. Axial (A, D, G), coronal (B, E, H), and sagittal (C, F, I) views illustrate representative outputs from the CERES (CEREbellum Segmentation) pipeline applied to a high-resolution T1-weighted magnetic resonance imaging (MRI) scan. Panels (A-C) depict lobular parcellation, with distinct colors corresponding to individual cerebellar lobules (I-X and Crus I-II) automatically delineated using the multi-atlas label fusion method. Panels (D-F) show PCT maps (mm; TIV^(1/3)^-normalized), highlighting local variations in cerebellar cortical thickness derived from inner-to-outer cortical surface distances. Panels (G-I) demonstrate tissue-type segmentation, distinguishing gray matter (red) and white matter (green) compartments for volumetric quantification. All segmentations were performed automatically by CERES without manual intervention, ensuring reproducibility and objectivity across participants. These outputs form the basis for volumetric, thickness, and asymmetry analyses in the present study, providing a fully standardized morphometric framework for evaluating cerebellar structure in adults with ASD.

Cerebellar morphometry was performed with the CERES module in the volBrain online platform, which provides fully automated, reproducible cerebellar segmentation from high-resolution T1-weighted MRI. CERES yields lobule-level measures of volume, PCT, and asymmetry using a validated multi-atlas patch-based label-fusion approach.^9^ Representative lobular boundaries and tissue segmentations from DeepCERES are shown in Figure 1.

After preprocessing, each hemisphere was parcellated into anatomically defined lobules (I-X and Crus I-II). Regional volumes were computed in cm^3^ and normalized to TIV. CERES also provided PCT estimates for each lobule; thickness values were derived in MNI152 space and normalized using the cubic root of TIV.^13^^,^^14^

### Asymmetry Indices Definition and Interpretation

Asymmetry indices (AI) were computed for each cerebellar lobule to identify lateralized anatomical patterns relevant to neurodevelopmental disorders. The AI was calculated using the formula:



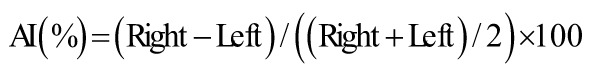

(1)


where positive values indicate rightward dominance and negative values indicate leftward dominance. This percent-normalized formulation is scale-invariant and reduces sensitivity to global head-size differences.^15^ To ensure methodological consistency, all AIs were derived from ComBat-harmonized volumetric values, minimizing scanner-related intensity and alignment biases across ABIDE sites. Asymmetry indices were subsequently incorporated into all group-comparison and sex-stratified analyses to evaluate potential diagnostic and biological relevance.

### Acquisition, Processing, and Quality Control

All morphometric parameters were exported by the volBrain/CERES pipeline in structured CSV format following the predefined participant selection described above. To preserve objectivity and reproducibility, no manual edits or post-processing interventions were applied to the outputs. Automated quality control (QC) procedures implemented within the volBrain platform were complemented by systematic visual inspection to identify potential motion artifacts, ghosting, misregistration, or segmentation failures.

The use of the CERES module ensured standardized lobular parcellation and cross-study comparability, building upon the automated preprocessing pipeline described earlier.^9^^,^^16^ All exported datasets underwent consistency checks prior to statistical analysis to verify correct hemispheric labeling, appropriate TIV normalization, and the absence of segmentation artifacts across cerebellar lobules.

Importantly, the definition of the analytic sample was fixed after completion of the initial ABIDE screening and QC procedures and prior to any statistical analysis. The final study cohort consisted of 48 adults with ASD (20 females, 28 males) and 52 HC participants (23 females, 29 males). No participants were added, excluded, or reclassified after this cohort definition, and all datasets included in the analyses satisfied both automated and visual QC criteria.

Sex-stratified analyses were performed by partitioning this predefined cohort into female and male subgroups based solely on biological sex, without applying any additional QC filters, lobule-specific exclusions, or region-dependent sample reductions. Thus, all global, regional, asymmetry, and sex-based analyses were conducted within the same fixed analytic sample.

### Alignment and Scanner Considerations

We explicitly considered two non-biological contributors to AI variance: (i) intra-/inter-scanner differences (pulse sequence, gradient nonlinearity, intensity profiles) and (ii) subtle head-alignment or registration imperfections. Even sub-voxel left–right misalignments can inflate or invert very small AIs (<1%-2%), particularly near high-curvature fissures and vermal borders.^15^ Additionally, site-specific contrast differences in multi-site datasets may influence hemispheric measurements.^11^ These issues were addressed through rigorous ComBat harmonization along with symmetry and registration quality checks performed prior to statistical analysis.

### Reliability Frame

In line with prior atlas-validation and scan–rescan studies, tiny AI magnitudes (~0.5%-1.5%) are interpreted as potentially reflecting test–retest or alignment variability, whereas medium-to-large AIs accompanied by convergent volume or thickness differences are more likely to represent true biological effects.^15^^,^^17^ Vermal and small lateral lobules are particularly susceptible to partial-volume and registration influences, which reinforces the focus on results that remain stable after multiplicity correction. Accordingly, only asymmetry effects exceeding the expected measurement-noise range were interpreted as biologically meaningful, consistent with the false discovery rate (FDR) corrected thresholds described in the Statistical Analysis section.

### Multi-site Harmonization

Given the aggregated multi-scanner cohort, AIs are reported together with FDR-adjusted significance and effect sizes, and acknowledge that residual site structure may still affect the smallest values. ComBat harmonization was used to reduce site-related variability while preserving biological differences across scanners.^11^ Additional safeguards included stringent QC of registration outputs, symmetry checks of left–right labels, and sensitivity analyses with alternative templates or warps.^18^^,^^19^ Together, these procedures strengthened confidence that observed asymmetry patterns reflected biological rather than methodological sources.

### Statistical Analysis

Statistical analyses were conducted using IBM SPSS Statistics for Windows, Version 21.0 (IBM Corp.; Armonk, NY, USA), R software, Version 4.3.3 (R Foundation for Statistical Computing; Vienna, Austria), and Python, Version 3.11 (Python Software Foundation; Wilmington, DE, USA). To address multi-site effects in ABIDE, ComBat harmonization was applied using group, sex, age, and TIV as biological covariates and Site/Scanner ID as batch effects, and results were checked against unharmonized data.^11^^,^^12^

Normality was assessed with Shapiro–Wilk; between-group comparisons used independent-samples *t*-tests or Mann–Whitney *U*-tests as appropriate, with effect sizes reported (Cohen’s *d*, *r*, partial η^2^). A general linear model tested group, sex, and group × sex effects adjusting for age and TIV. Multiple comparisons were controlled using FDR (q < 0.05) within predefined metric families (Supplementary Table 2). Correlations (Pearson/Spearman) used Bonferroni adjustment, and sex-stratified analyses were treated as exploratory.

## Results

This study primarily aimed to characterize sex-based morphometric patterns in the cerebellum of adults with ASD. However, to establish a baseline and provide context for subsequent sex-stratified comparisons, initial analyses were performed on the entire cohort to identify global ASD–HC group differences. These general effects are presented below, followed by sex-specific analyses that delineate how cerebellar alterations manifest differently in autistic males and females.

### Group Comparisons Between Autism Spectrum Disorder and Controls

Forty-eight adults with ASD and 52 matched controls were analyzed. Descriptive statistics for significant metrics appear in Table 1, with full non-significant outcomes in Supplementary Table 3. All values were normalized to TIV; however, TIV remained significantly larger in ASD (1527.9 ± 169.3 cm^3^ vs 1399.6 ± 137.5 cm^3^, *P* < .001), consistent with established brain overgrowth in autism. Significant cerebellar differences from parametric and non-parametric tests are listed in Tables 2-3. Parametric *t*-test outcomes for PCT measures and volumetric AI that remained significant after ComBat harmonization are provided in Supplementary Table 4 (including effect sizes and FDR-adjusted results).

### Global Cerebellar Architecture

**Figure 2:**
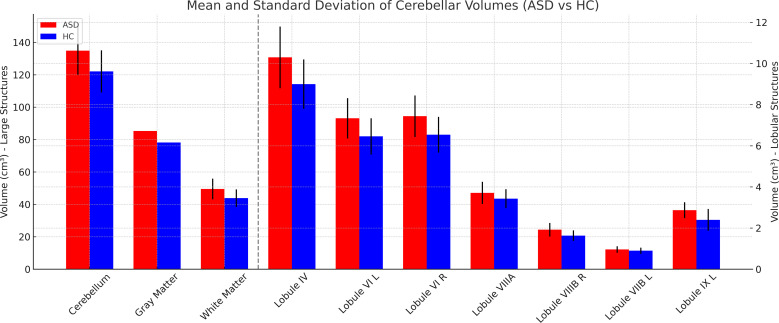
Volumetric comparisons of cerebellar macro- and micro-structural metrics between adults with autism spectrum disorder (ASD) and healthy controls (HC). These regions have been implicated in motor, executive, language, and socio-emotional functions in prior literature; however, the present cross-sectional dataset does not test behavior, clinical symptoms, or causal mechanisms. (A) Group-wise mean ± SD values for total cerebellar volume, total gray-matter volume, and total white-matter volume following ComBat harmonization. ASD participants (red bars) showed greater total cerebellar volume relative to HC (blue bars), primarily reflecting an increase in white-matter contribution rather than gray-matter expansion, in line with prior neurodevelopmental accounts; however, mechanistic interpretations (e.g., synaptic pruning) are not testable in the present cross-sectional design. (B) Lobule-specific volumetric differences for representative cerebellar subregions, including Lobule IV (sensorimotor), Lobule VI (executive–cognitive), Lobules VIIIA–B (posterior–cognitive), and Lobule IX (autonomic–limbic). Mean ± SD values (cm³) are displayed for each lobule across left, right, and total measures. Enlargements in anterior (Lobule IV) and executive (Lobule VI) territories have been implicated in motor-coordination and cognitive-planning domains, whereas posterior-lobular hypertrophy (VIIIA–B) may relate to social-behavioral modulation. The pronounced increase in vermal/limbic Lobule IX volume is conceptually consistent with autonomic-affective dysregulation in ASD; however, the present dataset does not test behavior or clinical symptoms. Error bars indicate ± 1 SD. All volumetric measures were normalized to total intracranial volume (TIV) to minimize head-size bias. Statistical comparisons were conducted on ComBat-harmonized datasets to reduce inter-site variability, using independent-samples *t*-tests and Mann–Whitney *U*-tests as appropriate. Multiple comparisons were controlled by the Benjamini–Hochberg false discovery rate (FDR) correction (*q* < 0.05). These results demonstrate that cerebellar enlargement in adults with ASD is regionally specific, with the strongest effects observed in sensorimotor and autonomic–limbic lobules.

The cerebellum was ~10% larger in ASD (134.9 ± 14.9 vs 122.1 ± 13.0 cm^3^, *P* < .001), with bilateral hemispheric enlargement (right +11%; left +10%) and increased gray (+9%) and white matter (+13%) volumes. White-matter effects were large (Cohen’s *d* = 1.32 right; 1.20 total; 1.04 left), while most lobular differences showed medium effects (*d* ≈ 0.55-0.70). Mann–Whitney results converged with parametric findings (*r* ≈ 0.45-0.48), confirming robust global enlargement (Figure 2). Macroscopic enlargement of both compartments is consistent with prior neurodevelopmental accounts proposing altered synaptic pruning and axonal development in ASD; however, given the cross-sectional adult design, these findings cannot adjudicate mechanism and should be interpreted as group-level morphometric differences that may relate to cerebello-cortical circuit efficiency.^2^

### Hemispheric and Tissue Asymmetry

ASD showed a rightward whole-cerebellum bias (+0.45 ± 1.84 %), opposite the leftward HC pattern (−0.63 ± 1.68 %, *P* = .004). White matter similarly shifted rightward (0.21 ± 1.08 % vs −0.39 ± 0.70 %, *P* < .001). Asymmetry effects were moderate-to-large (*d *≈ 0.59 for global AI; >0.8 for white matter), supported by medium Mann–Whitney effects (*r* = 0.436).

### Sensorimotor Territory (Lobules III–V)

Lobule IV volume and thickness were significantly lower in ASD (both *P* < .001), with large effects (*d* ≈ 0.94-0.97). Lobule V exhibited a marked asymmetry reversal: ASD showed leftward bias (−2.2 ± 9.3 %) vs strong rightward dominance in HC (+5.6 ± 6.2 %, *P* < .001), a large effect (*d* ≈ 0.85) supported by medium non-parametric effects (*r* = 0.431).

### Executive Hub (Lobule VI)

Lobule VI was bilaterally reduced (*P* = .049), with small-to-medium volume effects (*d* ≈ 0.40) and a consistent leftward asymmetry shift supported by medium Mann–Whitney values (*r* = 0.431).

### Posterior Cognitive Circuitry (Lobules VIIIA–B)

Lobule VIIIA showed a lower mean volume in ASD than controls (ASD total: 3.015 ± 0.276 vs HC: 3.206 ± 0.296 cm^3^; *P* = .001), with a leftward shift in asymmetry (ASD: −0.016 ± 6.633% vs HC: +1.260 ± 5.025%). Effects were medium for VIIIA (*d* ≈ 0.67) and small-to-medium for VIIIB thickness (*d* ≈ 0.40), with convergent Mann–Whitney effects (*r* ≈ 0.39-0.43).

### Temporal–Language Territory (Lobule VIIB)

Left Lobule VIIB volume was modestly lower in ASD (ASD: 3.194 ± 0.284 vs HC: 3.319 ± 0.312 cm^3^, *P* = .039) with a small-to-medium effect (*d* ≈ 0.42).

### Autonomic–Limbic Vermis (Lobule IX)

Vermal Lobule IX showed the strongest deviations, with increased left-hemisphere volume (ASD: 2.873 ± 0.386 vs HC: 2.395 ± 0.528 cm^3^) and a pronounced leftward asymmetry shift (ASD: −6.801 ± 7.880% vs HC: +6.315 ± 9.127%). Effect sizes exceeded *d* = 0.90, and non-parametric effects were large (*r* = 0.651), confirming Vermal IX as a prominent group-level neuroanatomical feature.

### White-Matter Hypertrophy and Asymmetry

White matter was 13% larger in ASD (49.5 ± 6.4 vs 43.9 ± 5.4 cm^3^, *P* < .001), with the dominant effect in the right hemisphere. Effect sizes were very large (*d* = 1.32 right; 1.20 total; 1.04 left), suggesting a robust between-group difference at the group level.

Overall, ASD cerebellar morphology showed global enlargement (notably in cerebellar white matter), alongside regionally specific increases (e.g., vermal lobule IX) and decreases (e.g., lobule IV and selected posterior lobules), and consistent asymmetry shifts—suggesting a reorganized topography that can be discussed within established functional frameworks; however, the present dataset does not test behavior or clinical symptoms.

### Group Comparisons Between Females and Males

Sex-stratified analyses were performed within the predefined study cohort. The ASD group consisted of 48 adults, including 20 females and 28 males, while the HC group comprised 52 adults (23 females and 29 males). No additional exclusions or sample redefinitions were applied for sex-based comparisons, and all analyses were conducted within this fixed analytic sample.

For descriptive purposes, subgroup-level age distributions are reported to contextualize sex-specific findings. Autistic women had a mean age of 26.9 ± 5.2 years, and neurotypical women had a mean age of 27.3 ± 4.9 years. Autistic men had a mean age of 25.8 ± 4.7 years, while neurotypical men had a mean age of 26.4 ± 5.0 years. All cerebellar morphometric measures were normalized to TIV.

### Female Comparison

**Figure 3:**
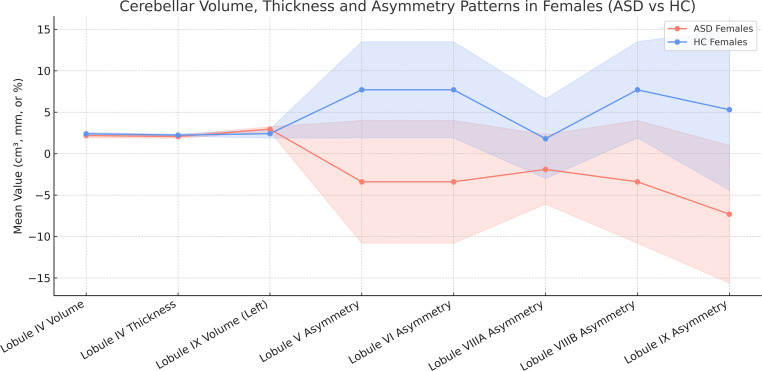
Sex-specific cerebellar morphometric alterations in females with autism spectrum disorder (ASD) compared with healthy controls (HC). Line plots with shaded error bands (mean ± SD) illustrate cerebellar volume (cm³, total intracranial volume [TIV]-normalized), pseudo-cortical thickness (PCT) (mm, TIV^(1/3)-normalized), and asymmetry indices for key lobules among autistic and neurotypical females (ASD: n = 20; HC: n = 23). Autistic women (red) exhibit a distinct morphometric profile characterized by bilateral reduction of Lobule IV, left-dominant enlargement of Lobule IX, and inversions of asymmetry direction across sensorimotor (Lobule V), executive (Lobule VI), posterior-cognitive (Lobules VIIIA–B), and autonomic–limbic (Lobule IX) regions relative to controls (blue). These polarity reversals indicate a leftward shift in cerebellar lateralization, replacing the normative rightward bias observed in HC females. Because these lobules have been implicated in fine-motor timing, salience-network regulation, and autonomic integration, the observed pattern may be relevant to reported sex-differential phenotypes in ASD; no direct behavioral associations were tested, and the interpretation is therefore hypothesis-generating. All measures were derived from ComBat-harmonized datasets normalized to TIV, and statistical significance was evaluated using false discovery rate (FDR)-corrected non-parametric tests (*q* < 0.05).

Group comparisons of regional cerebellar volume, cortical thickness, and asymmetry between females with ASD and HC are presented in Table 4, using Mann–Whitney *U*-tests. These comparisons include *P* values, U statistics, mean group differences, and CI. The corresponding patterns are also visualized in Figure 3. Detailed female-subgroup Mann–Whitney U results for significant volumetric, PCT, and asymmetry outcomes (including Z statistics, effect direction, CI, and FDR control within lobular domains) are provided in Supplementary Table 5.

### Anterior Sensorimotor Lobe (Lobules IV–V)

Autistic women showed a bilateral reduction of Lobule IV volume (2.18 ± 0.27 cm³ vs 2.39 ± 0.17 cm³) together with reduced mean thickness, indicating concurrent volumetric and thickness alterations in this anterior sensorimotor lobule. In contrast, Lobule V asymmetry was inverted: ASD women demonstrated a left‑dominant bias of ‑3.4 ± 7.4 % compared with a strong rightward bias of +7.7 ± 5.8 % in controls (*P* < .001).

### Transitional Lobule VI

Both volumetric and thickness asymmetries of Lobule VI shifted from rightward in controls to leftward in ASD women (*P* < .001), indicating a consistent directional change in lateralization within this lobule.

### Posterior Cognitive Lobules (VIIIA–VIIIB)

Left‑ward shifts were also observed in posterior lobular asymmetries (VIIIA ‑1.9 ± 4.2 % vs +1.8 ± 4.8 %) and in Lobule VIIIB (*P* < .001), supporting a broader pattern of altered posterior lateralization in ASD women.

### Autonomic–Limbic Lobule IX

ASD females exhibited a marked enlargement of the left Lobule IX (2.96 ± 0.29 cm³ vs 2.41 ± 0.53 cm³, *P* = .003) accompanied by a strong negative asymmetry (‑7.3 ± 8.3 % vs +5.3 ± 9.7 %).

### Male Comparison

**Figure 4.**
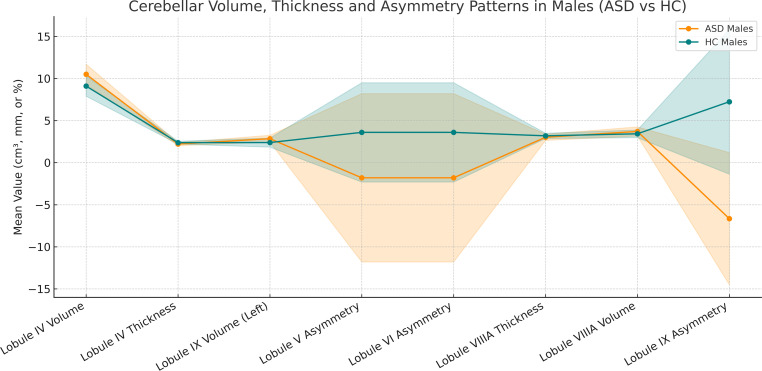
Sex-specific cerebellar morphometric alterations in males with autism spectrum disorder (ASD) compared with healthy controls (HC). Line plots with shaded error bands (mean ± SD) depict cerebellar volume (cm³, total intracranial volume [TIV]-normalized), pseudo-cortical thickness (PCT) (mm, TIV^(1/3)-normalized), and asymmetry index distributions across representative lobules in autistic (orange) and neurotypical (green-blue) males (ASD: n = 28; HC: n = 29). In contrast to the female pattern, autistic men demonstrated a more generalized enlargement of cerebellar white matter and subtle posterior lobular thinning, particularly in Lobules VIIIA–B, coupled with reduced leftward asymmetry in Lobules V and VI and an enhanced rightward bias in Lobule IX. This configuration suggests a divergent neuroanatomical lateralization profile between sexes, where males show attenuated inter-hemispheric differentiation and posterior hypoplasia rather than anterior hypertrophy. Given that these territories have been implicated in motor, repetitive behavior, and executive timing networks in prior work, the observed profile is discussed within that conceptual framework; however, no direct behavioral associations were tested in the present dataset. All parameters were derived from ComBat-harmonized and TIV-normalized datasets, with between-group contrasts evaluated by non-parametric tests and corrected for multiple comparisons using the Benjamini–Hochberg false discovery rate (FDR, *q* < 0.05).

Cerebellar volume, thickness, and asymmetry comparisons between males with ASD and HC are presented in Table 5, based on Mann–Whitney *U*-tests, including significance levels. The corresponding structural patterns are illustrated in Figure 4, supporting the statistical findings. Detailed male-subgroup Mann–Whitney U results for significant PCT and volume-based asymmetry outcomes (including Z statistics, CI, and FDR control within lobular domains) are provided in Supplementary Table 6.

### Global Cerebellar Volumes and Tissue Composition

Autistic men displayed a 9.6% increase in total cerebellar volume (139.45 ± 10.50 cm³) relative to neurotypical men (127.27 ± 11.41 cm³, *P* < .001). Grey matter was 8% larger (88.38 ± 7.46 vs 81.51 ± 8.11 cm³), whereas white matter showed a striking 12% expansion (51.07 ± 3.66 vs 45.76 ± 3.62 cm³).

### Hemispheric Asymmetry

Right‑hemisphere white matter predominated in ASD men (25.71 ± 2.03 vs 22.65 ± 1.82 cm³, *P* < .001), whereas controls retained a subtle leftward balance.

### Lobules III–V: Fine–Motor Gateway

As in females, Lobule V asymmetry inverted from rightward to leftward (volume ‑1.8 ± 10.0 % vs +3.6 ± 5.9 %, *P* < .001). The parallel shift in thickness suggests a genuine architectural rebalancing rather than measurement noise.

### Lobule VI and Crus I/II -Complex

Volume and thickness asymmetries of Lobule VI likewise flipped polarity, reinforcing the notion that sex‑convergent structural reorganization targets the sensorimotor‑executive transition zone.

### Posterior Lobules VIIIA/B and VIIB

Unlike females, autistic men showed subtle bilateral thinning of Lobule VIIIA (right‑hemisphere thickness 3.02 ± 0.37 vs 3.19 ± 0.31 mm; *P* = .043), suggesting hypo‑development rather than hypertrophy.

### Vermal Lobule IX

The most prominent male‑specific anomaly was a mirror image of the female pattern: both volume and thickness asymmetry of Lobule IX shifted dramatically toward the left (‑6.65 ± 7.85 % vs +7.24 ± 8.60 %, *P* < .001), with absolute left‑hemisphere enlargement (2.85 ± 0.41 vs 2.39 ± 0.54 cm³).

Despite clear sex differences in the topography of cerebellar alterations, a unifying motif emerged: autistic participants of both sexes exhibited systematic leftward shifts in lobular asymmetry that replaced the normative rightward bias.

### Robustness Analysis

FDR-adjusted analyses confirmed the stability of the main findings: several group-level effects remained highly significant after correction—most prominently the AI of lobules V, VI, VIIIB, and IX (e.g., Lobule IX Volume Asymmetry: F(1,95)=17.53, *P *< .0001, *q* = 0.0012, partial η^2^ = 0.156) and the left-sided thickness and volume of Lobule IX (F(1,95) = 10.28, *P* = .0018, *q *= 0.0198, partial η^2^ = 0.098). These moderate-to-large effects indicate biologically meaningful and replicable differences rather than statistical noise, and their preservation despite multi-site sampling strengthens their potential as ASD-related group-level distinguishing features.

Group × sex interactions showed nominal signals in left lobules V, VI, and VIIIB (all q≈0.97) but did not survive correction. Nevertheless, the effect directions matched the a priori hypotheses and partial η^2^ values (≈0.02-0.03) suggest small yet coherent sex-linked tendencies that may emerge more clearly in larger or prospectively harmonized datasets. Collectively, these results refine the interpretation of the primary findings and highlight the cerebellar regions most likely to represent stable ASD signatures.

### Effects of ComBat Harmonization

ComBat harmonization reduced site/scanner variance across the 4 ABIDE sites, and all ASD–HC comparisons were conducted on the harmonized dataset. Effect directions were preserved for all 59 cerebellar variables. No FDR-significant finding was lost, and 14 additional parameters reached significance (q < 0.05), mainly in anterior and posterior sensorimotor territories (III–V and VI–VIIB), consistent with near-threshold trends in the unharmonized data. Harmonization changed effect-size magnitudes without reversing direction, supporting improved precision through removal of non-biological variance. The largest effects remained in vermal lobule IX and right cerebellar white matter (*d* > 2.0). Full pre/post-harmonization statistics are provided in Supplementary Table 2.

## Discussion

### Global Cerebellar Alterations in Autism Spectrum Disorder

In this study, adults with ASD showed robust cerebellar morphometric alterations relative to HC, confirming and extending prior work on cerebellar contributions to autism. Prior meta-analyses and imaging studies consistently report larger cerebellar volumes in autism, which is consistent with atypical neurodevelopmental trajectories; however, mechanistic explanations such as reduced synaptic pruning or altered white-matter maturation remain speculative in an adult, cross-sectional sample.^6^^,^^20^^,^^21^ Effect sizes were large for global metrics (*d* > 1.0) and medium-to-large for most lobular measures (*d* ≈ 0.55-0.97), underscoring the biological significance of these findings. These values indicate that the observed structural differences are not only statistically significant but also biologically meaningful at the group level, supporting their relevance as reproducible group-level neuroanatomical features; however, they should not be interpreted as clinically actionable or diagnostically informative at the individual level without behavioral/clinical correlation and external replication.

### Altered Cerebellar Asymmetry Patterns

Similar results have been documented by Stoodley and Schmahmann,^22^ who proposed that deviations in cerebellar lateralization—whether reflecting altered hemispheric organization, compensatory reweighting, or developmental divergence—may contribute to atypical cerebro-cerebellar circuitry in ASD.^25^ Effect size analysis indicated a moderate magnitude (*d* ≈ 0.59), suggesting a systematic group-level difference in the asymmetry index; however, whether this reflects lateralization, compensation, or other organizational factors cannot be determined without behavioral correlates and longitudinal data. Accordingly, this finding should be interpreted cautiously as a group-level lateralization difference rather than as evidence of compensation or functional impairment.

### Region-Specific Structural Differences

Region-specific analyses localized the strongest alterations to sensorimotor and executive territories. Lobules IV–V showed pronounced alterations, including robust reductions in Lobule IV volume (large effects) and a marked asymmetry reversal in Lobule V. These anterior cerebellar differences have been implicated in motor timing and coordination in prior work; however, direct links to fine-motor performance cannot be inferred here because behavioral measures were not modeled in the present cross-sectional sample.^21^^,^^23^ The asymmetry reversal observed in Lobule V—from a typical rightward dominance in controls to leftward dominance in ASD—aligns with reports of atypical cerebellar lateralization in ASD; nevertheless, any functional interpretation remains hypothesis-generating in the absence of direct behavioral correlations.^23^

Lobule VI showed a modest bilateral volume reduction together with a consistent leftward asymmetry shift (small-to-moderate effects), in line with prior literature implicating lobule VI within executive–cognitive networks in ASD.^7^^,^^24^ These group-level differences should not be interpreted as evidence of a specific executive deficit in the present dataset. Posterior lobules VIIIA–B and VIIB exhibited volume reductions of small-to-medium magnitude, consistent with reports of cerebellar involvement in distributed cognitive and social-motor networks; however, structure–symptom interpretations remain indirect here without behavioral measures.^7^ Vermal lobule IX demonstrated the largest and most consistent alterations (*d* > 0.90), aligning with evidence implicating this autonomic–limbic region in stress-related regulation differences frequently discussed in ASD;^25^ nonetheless, clinical interpretations remain speculative without direct physiological or symptom-level measures.

Thus, the findings provide additional group-level anatomical evidence implicating these lobules in ASD-related cerebellar variation, while any linkage to social synchronization or repetitive behaviors should be regarded as tentative and requires dedicated behavioral and multimodal validation.

### Sex-Specific Morphometric Patterns

Sex-stratified analyses suggested that cerebellar morphometric differences may vary by sex; however, these subgroup patterns should be interpreted as exploratory given the modest subgroup sizes and the absence of FDR-significant group-by-sex interaction effects. Female ASD subjects exhibited subgroup differences in volume and AI in anterior and autonomic–limbic regions; these patterns are exploratory and should not be interpreted as reflecting internalizing behaviors or motor impairment without formal clinical/behavioral modeling. Conversely, autistic males demonstrated broader white matter differences and subtle posterior lobular thickness/asymmetry findings; while prior literature has discussed possible behavioral relevance, any structure–symptom interpretation remains indirect here and should be considered hypothesis-generating^14^^,^^13^^,^^26^

### Methodological Considerations and Reliability of Asymmetry Metrics

Methodologically, the CERES pipeline ensured high segmentation reliability.^9^^,^^16^ Nonetheless, cerebellar AI remains susceptible to minor registration differences and scanner heterogeneity; therefore, the interpretations prioritized effects that survived FDR correction, exhibited medium-to-large effect sizes, and converged across analytic approaches. ComBat harmonization substantially reduced site-related variance while preserving biological differences, stabilizing AI estimates and strengthening confidence in subtle asymmetry signals.^11^

### FDR-Corrected Features and Clinical Interpretation

FDR-adjusted models identified the most reproducible features: AI of lobules V, VI, VIIIB, and IX, and left-lateralized Lobule IX volume/thickness (q ≤ 0.02), with partial η^2^ approaching 0.15.^6^^,^^27^ These effect-size patterns, coupled with cross-method convergence, reinforce the robustness of the surviving lobular differences and suggest that they reflect stable group-level neuroanatomical characteristics of ASD, without implying specific functional or clinical correlates. The persistence of these signals despite multi-site acquisition heterogeneity underscores their interpretation as reproducible group-level neuroanatomical features rather than scanner-specific artifacts. Accordingly, these findings are framed as group-level neuroanatomical signs of ASD rather than diagnostic or prognostic indicators, and no clinical utility is proposed without prospective replication and behavioral validation. These territories have been implicated in fine-motor timing, autonomic regulation, and executive–salience processing in prior work; therefore, the observed group-level structural differences can be discussed in relation to these domains only as a conceptual framework, not as evidence of specific motor, autonomic, or cognitive deficits in this sample.^28^^,^^29^ Directional consistency of group × sex trends, despite q ≈ 0.97, further implies meaningful but low-magnitude sex effects.

### Impact of ComBat Harmonization on Robustness of Cerebellar Findings

Harmonization effectively minimized scanner-related variance while preserving group differences.^11^^,^^12^ All pre-harmonization significant results remained significant post-harmonization, and 14 additional endpoints emerged—primarily within anterior (III–V) and posterior sensorimotor regions (VI–VIIB)—indicating that multi-site noise had masked spatially coherent but subtle alterations. The amplification of effects in vermal IX and cerebellar white matter further supports their status as biologically stable rather than scanner-dependent findings.^27^^,^^30^

Asymmetry metrics demonstrated directional stability across harmonization, supporting the interpretation that ComBat reduced dispersion without generating spurious lateralization.^11^ Nonetheless, asymmetry measures inherently magnify small alignment errors, so convergent volume–thickness–AI results within the same lobules were used as a robustness criterion.^2^^,^^28^ From a clinical standpoint, this configuration is compatible with domains often discussed in adult ASD (e.g., motor planning/timing and attention–affect coupling), but this study does not test structural–behavioral associations; thus, any phenotypic interpretation should remain cautious and hypothesis-generating.^7^ The persistence of very large effects in vermal IX and right cerebellar white matter after harmonization supports their interpretation as reproducible group-level neuroanatomical features rather than scanner-specific artifacts.^27^^,^^30^

### Limitations and Considerations

Limitations include the relatively modest overall sample, and especially the small sex-specific subgroups, which represent a major constraint that may substantially reduce statistical power and restrict the stability and generalizability of sex-stratified findings. First, the cross-sectional design precludes inference on developmental trajectories; longitudinal studies are needed to determine how these cerebellar alterations evolve and how they may relate to clinical outcomes. Second, reliance on structural MRI limits functional interpretation; incorporating diffusion and functional imaging may better link anatomical variation with behavioral phenotypes. Third, although ABIDE provides behavioral indices such as ADOS, incomplete coverage prevented their inclusion to avoid bias and loss of power. Additional considerations include the statistical assumptions inherent to ComBat (e.g., distributional form, covariate specification) and the sensitivity of asymmetry metrics to segmentation nuances. Larger, balanced multi-site datasets with richer phenotyping and complementary imaging modalities will be essential to validate these findings and clarify their developmental and functional significance.

## Conclusion

This study suggests that adults with ASD show a consistent cerebellar signature characterized by regional hypertrophy and altered lateralization, identified through high-resolution CERES morphometry. By utilizing the automated CERES segmentation tool,the region-specific volumetric enlargement and altered lateralization were observed across multiple cerebellar territories. These findings support the view that the cerebellum is meaningfully involved in ASD-related neuroanatomical variation beyond the traditional motor domain, while causal or clinical inferences cannot be drawn from this cross-sectional dataset. Accordingly, these cerebellar differences are interpreted as group-level neuroanatomical signs rather than group-level distinguishing features, and clinical applicability is not yet evidence-based in the absence of replication and direct symptom-level validation. Importantly, sex-stratified results are presented as exploratory and require replication in larger, balanced cohorts with formal group×sex interaction testing. By clarifying group-level neuroanatomical patterns potentially related to ASD-relevant domains, such studies may guide future research; however, diagnostic precision and targeted interventions would require prospective clinical validation with standardized behavioral measures. Ultimately, the present work contributes to a growing body of anatomical evidence suggesting that the cerebellum may be integrally involved in ASD and underscores the value of high-resolution, automated neuroimaging tools in advancing the understanding of neurodevelopmental disorders.

## Supplementary Materials

Supplementary Material

## Figures and Tables

**Table 1.** Descriptive Statistics of Cerebellar Volumetric Measures in Adults with ASD and HC

**Variable**	**Group**	n	**Mean**	**SD**	**Minimum**	**Maximum**
Cerebellum_Volume_Left	ASD	48	67.266	7.352	42.630	80.690
Cerebellum_Volume_Left	HC	52	61.250	6.511	43.480	76.510
Cerebellum_Volume_Right	ASD	48	67.610	7.640	41.180	81.700
Cerebellum_Volume_Right	HC	52	60.879	6.575	43.590	76.460
Cerebellum_Volume_Total	ASD	48	134.874	14.941	83.810	162.390
Cerebellum_Volume_Total	HC	52	122.127	13.043	87.080	152.970
Gray Matter_Volume_Left	ASD	48	42.658	5.259	23.760	52.510
Gray Matter_Volume_Left	HC	52	39.039	4.359	27.170	49.360
Gray Matter_Volume_Right	ASD	48	42.680	5.245	23.970	52.300
Gray Matter_Volume_Right	HC	52	39.148	4.508	27.620	50.230
Gray Matter_Volume_Total	ASD	48	85.338	10.452	47.730	104.810
Gray Matter_Volume_Total	HC	52	78.187	8.833	54.790	99.590
Lobule III_Volume_Left	ASD	48	1.763	0.347	0.740	2.840
Lobule III_Volume_Left	HC	52	1.592	0.263	0.960	2.170
Lobule III_Volume_Right	ASD	48	1.804	0.354	1.080	2.880
Lobule III_Volume_Right	HC	52	1.649	0.326	0.580	2.330
Lobule III_Volume_Total	ASD	48	1.785	0.331	1.010	2.860
Lobule III_Volume_Total	HC	52	1.619	0.278	0.780	2.210
Lobule IV_Volume_Left	ASD	48	2.156	0.257	1.560	2.630
Lobule IV_Volume_Left	HC	52	2.336	0.185	1.880	2.790
Lobule IV_Volume_Right	ASD	48	2.178	0.233	1.660	2.710
Lobule IV_Volume_Right	HC	52	2.374	0.187	1.960	2.670
Lobule IV_Volume_Total	ASD	48	2.167	0.220	1.720	2.570
Lobule IV_Volume_Total	HC	52	2.355	0.171	1.920	2.730
Lobule IX_Volume_Left	ASD	48	2.873	0.386	1.670	3.560
Lobule IX_Volume_Left	HC	52	2.395	0.528	1.360	3.410
Lobule IX_Volume_Right	ASD	48	2.691	0.411	1.680	3.240
Lobule IX_Volume_Right	HC	52	2.536	0.486	1.540	3.390
Lobule IX_Volume_Total	ASD	48	2.782	0.385	1.680	3.370
Lobule IX_Volume_Total	HC	52	2.466	0.498	1.450	3.350
Lobules I-II_Volume_Left	ASD	48	1.660	0.945	0.280	4.050
Lobules I-II_Volume_Left	HC	52	1.698	0.772	0.450	4.100
Lobules I-II_Volume_Right	ASD	48	1.737	0.950	0.420	3.970
Lobules I-II_Volume_Right	HC	52	1.799	0.747	0.520	4.160
Lobules I-II_Volume_Total	ASD	48	1.699	0.943	0.400	4.010
Lobules I-II_Volume_Total	HC	52	1.749	0.754	0.480	4.130
Lobule VIIB_Volume_Left	ASD	48	3.194	0.284	2.530	4.080
Lobule VIIB_Volume_Left	HC	52	3.319	0.312	2.750	4.060
Lobule VIIB_Volume_Right	ASD	48	3.242	0.284	2.620	4.310
Lobule VIIB_Volume_Right	HC	52	3.323	0.339	2.600	4.120
Lobule VIIB_Volume_Total	ASD	48	3.218	0.267	2.640	4.190
Lobule VIIB_Volume_Total	HC	52	3.321	0.314	2.670	4.090
Lobule VIIIA_Volume_Left	ASD	48	3.016	0.291	2.330	3.730
Lobule VIIIA_Volume_Left	HC	52	3.185	0.300	2.360	3.730
Lobule VIIIA_Volume_Right	ASD	48	3.015	0.294	2.410	3.820
Lobule VIIIA_Volume_Right	HC	52	3.226	0.314	2.540	3.870
Lobule VIIIA_Volume_Total	ASD	48	3.015	0.276	2.490	3.770
Lobule VIIIA_Volume_Total	HC	52	3.206	0.296	2.500	3.800
Lobule VIIIB_Volume_Left	ASD	48	3.043	0.232	2.510	3.550
Lobule VIIIB_Volume_Left	HC	52	3.020	0.269	2.170	3.480
Lobule VIIIB_Volume_Right	ASD	48	2.983	0.284	2.150	3.710
Lobule VIIIB_Volume_Right	HC	52	3.192	0.259	2.380	3.720
Lobule VIIIB_Volume_Total	ASD	48	3.013	0.222	2.530	3.560
Lobule VIIIB_Volume_Total	HC	52	3.107	0.248	2.270	3.600
Lobule X_Volume_Left	ASD	48	3.699	0.350	2.820	4.570
Lobule X_Volume_Left	HC	52	3.658	0.306	2.700	4.100
Lobule X_Volume_Right	ASD	48	3.734	0.359	2.740	4.350
Lobule X_Volume_Right	HC	52	3.635	0.263	3.090	4.120
Lobule X_Volume_Total	ASD	48	3.716	0.329	2.980	4.460
Lobule X_Volume_Total	HC	52	3.646	0.238	3.040	4.080
White Matter_Volume_Left	ASD	48	24.608	2.359	18.630	28.900
White Matter_Volume_Left	HC	52	22.211	2.307	16.310	27.150
White Matter_Volume_Right	ASD	48	24.928	2.630	17.210	29.900
White Matter_Volume_Right	HC	52	21.729	2.266	15.970	26.230
White Matter_Volume_Total	ASD	48	49.537	4.916	36.080	58.800
White Matter_Volume_Total	HC	52	43.940	4.486	32.280	53.380

This table presents descriptive statistics for global and lobular cerebellar volumetric measures in adults with ASD and HC. For each variable, group-specific sample size (N), mean, SD, minimum, and maximum values are reported. All volumetric measures are expressed in cm^3^ and normalized to TIV to account for inter-individual differences in head size. Aautism spectrum disorder and HC values are presented consecutively for each variable to facilitate direct comparison. No statistical inference is reported in this table.

ASD, autism spectrum disorder; HC, healthy control; TIV, total intracranial volume; SD, standard deviation.

**Table 2.** Independent-Samples *t*-test Results for Cerebellar Volumetric Measures Showing Significant Between-Group Differences After ComBat Harmonization

**Variable**	**F**	***t***	**Cohen’s *d***	**Interpretation**	***df***	***P***	**MD**	**SE**	**CI Lower**	**CI Upper**
White Matter_Volume_Right	0.232	6.531	**1.32**	Large effect	98	.000	3.199	0.490	2.227	4.171
White Matter_Volume_Total	0.016	5.953	**1.20**	Large effect	98	.000	5.596	0.940	3.731	7.462
WhiteMatter_Volume_Left	0.007	5.134	**1.04**	Large effect	98	.000	2.397	0.467	1.470	3.323
LobuleIV_Volume_Total	3.483	−4.786	**−0.97**	Large effect	98	.000	−0.188	0.039	−0.266	−0.110
LobuleIV_Volume_Right	1.875	−4.652	**−0.94**	Large effect	98	.000	−0.196	0.042	−0.279	−0.112
LobuleIV_Volume_Left	5.891	−4.046	**−0.82**	Large effect	98	.000	−0.180	0.045	−0.268	−0.092
LobuleVIIIA_Volume_Right	0.541	−3.462	**−0.70**	Medium effect	98	.001	−0.211	0.061	−0.332	−0.090
LobuleVIIIA_Volume_Total	0.596	−3.327	**−0.67**	Medium effect	98	.001	−0.191	0.057	−0.305	−0.077
LobuleVIIIA_Volume_Left	0.145	−2.850	**−0.58**	Medium effect	98	.005	−0.169	0.059	−0.286	−0.051
LobuleIII_Volume_Left	3.008	2.800	**0.57**	Medium effect	98	.006	0.172	0.061	0.050	0.294
LobuleIII_Volume_Total	1.471	2.724	**0.55**	Medium effect	98	.008	0.166	0.061	0.045	0.287
LobuleIII_Volume_Right	0.834	2.278	**0.46**	Small effect	98	.025	0.155	0.068	0.020	0.290
LobuleVIIB_Volume_Left	1.283	−2.089	**−0.42**	Small effect	98	.039	−0.125	0.060	−0.243	−0.006
LobuleVIIIB_Volume_Total	0.334	−1.996	**−0.40**	Small effect	98	.049	−0.094	0.047	−0.188	−0.001

This table presents parametric independent-samples *t*-test results for cerebellar lobular volumetric measures that showed significant differences between adults with ASD and neurotypical controls. Reported statistics include Levene’s test for equality of variances (*F*), *t* value, degrees of freedom (*df*), *P* value, mean difference (MD), standard error (SE), 95% CI, and Cohen’s *d* as an effect-size indicator. All volumetric measures were expressed in cm^3^ and normalized to TIV to account for inter-individual differences in head size. Scanner- and site-related variability was minimized using ComBat harmonization with sex, age, and TIV included as covariates. Multiple comparisons were controlled within functionally defined cerebellar lobular domains using the Benjamini–Hochberg FDR (*q* < 0.05).

ASD, autism spectrum disorder; FDR, false discovery rate; HC, healthy control; MD, mean difference; SE, standard error; TIV, total intracranial volume.

**Table 3.** Mann–Whitney *U*-test Results for Cerebellar Volumetric Measures and Derived AI Showing Significant Group Differences (ASD vs. HC) After ComBat Harmonization

**Variable**	**Mann–Whitney *U***	**Wilcoxon** ***W***	***Z***	**Effect size** **(*r*)**	**Interpretation**	*P*	**MD**	**SE**	**CI Lower**	**CI Upper**
Cerebellum_Volume_Left	597	1975	−4.491	0.449	Medium effect	.000	6.016	1.393	3.285	8.746
Cerebellum_Volume_Right	549	1927	−4.823	0.482	Medium effect	.000	6.731	1.431	3.926	9.536
Cerebellum_Volume_Total	568	1946	−4.692	0.469	Medium effect	.000	12.747	2.815	7.230	18.264
Gray Matter_Volume_Left	648	2026	−4.140	0.414	Medium effect	.000	3.618	0.970	1.716	5.520
Gray Matter_Volume_Right	652	2030	−4.112	0.411	Medium effect	.000	3.532	0.982	1.608	5.457
Gray Matter_Volume_Total	653	2031	−4.102	0.410	Medium effect	.000	7.150	1.943	3.341	10.959
Lobule IX_Volume_Left	566	1944	−4.706	0.471	Medium effect	.000	0.478	0.092	0.298	0.658
Lobule IX_Volume_Total	781	2159	−3.223	0.322	Medium effect	.001	0.316	0.089	0.142	0.490
Lobule VIIIB_Volume_Right	682	1858	−3.906	0.391	Medium effect	.000	−0.210	0.054	−0.316	−0.103
White Matter_Volume_Asymmetry	616	1994	−4.360	0.436	Medium effect	.000	0.605	0.139	0.332	0.878

This table summarizes the results of non-parametric Mann–Whitney *U* analyses applied to cerebellar volumetric measures and derived AI that violated normality assumptions. Reported metrics include Mann–Whitney *U*, Wilcoxon *W*, *Z* statistic, effect size (*r*), effect interpretation, *P* value, mean difference (MD), standard error (SE), and 95% CI. All cerebellar volumetric measures were expressed in cm^3^ and normalized to TIV. Site/scanner effects were controlled using ComBat harmonization (covariates: sex, age, TIV). Multiple comparisons were corrected with Benjamini–Hochberg FDR (*q* < 0.05) within functional lobular domains to ensure rigorous type I error control. The asymmetry index reflects relative hemispheric differences derived from volumetric measures and is inherently scale independent.

ASD, autism spectrum disorder; CI, confidence interval; FDR, false discovery rate; HC, healthy control; SE, standard error; TIV, total intracranial volume; *U*, Mann–Whitney statistic; *W*, Wilcoxon rank-sum test; *Z*, standardized score.

**Table 4.** Sex-Stratified Group Comparisons of Cerebellar Volumetric Measures Between Females with ASD and Healthy Controls (*P* < .05)

**Variable**	**ASD** **Mean**	**HC** **Mean**	***U***	***W***	***Z***	*P*	**SE**	**CI** **Lower**	**CI** **Upper**
Lobule IX_Volume_Left	2.964	2.406	223	289	2.953	.003	0.138	0.287	0.829
Lobule IV_Volume_Right	2.178	2.416	63	129	−2.541	.012	0.090	−0.414	−0.063
Lobule IX_Volume_Total	2.864	2.466	205	271	2.318	.021	0.138	0.127	0.668
Lobule IV_Volume_Total	2.182	2.392	73	139	−2.215	.028	0.089	−0.384	−0.036

This table presents subgroup analyses restricted to female participants. Non-parametric Mann–Whitney *U*-test results are reported for volumetric cerebellar measures showing significant differences between females with ASD and neurotypical females (ASD: n = 20; HC: n = 23). The table includes *U*, *W*, *Z*, *P* value, SE, and 95% CI values. All volumetric measures, including reported group differences and CI, were expressed in cm^3^ and normalized to TIV and corrected for multiple comparisons using the Benjamini–Hochberg false discovery rate (FDR; *q* < 0.05) within functional lobular domains. Site/scanner variance was mitigated through ComBat harmonization applied to the full dataset (covariates: age, TIV).

ASD, autism spectrum disorder; CI, confidence interval; FDR, false discovery rate; HC, healthy control; SE, standard error; TIV, total intracranial volume; *U*, Mann–Whitney statistic; *W*, Wilcoxon rank-sum test; *Z*, standardized score.

**Table 5.** Sex-Stratified Group Comparisons of Cerebellar Volumetric Measures Between Males with ASD and HC (*P* < .05)

**Variable**	**ASD** **Mean**	**HC** **Mean**	***U***	***W***	***Z***	*P*	**SE**	**CI Lower**	**CI Upper**
Cerebellum_Volume_Left	69.522	63.689	775	1478	3.745	.000	1.413	3.063	8.603
WhiteMatter_Volume_Left	25.359	23.106	792	1495	3.983	.000	0.472	1.329	3.178
WhiteMatter_Volume_Right	25.710	22.652	867	1570	4.996	.000	0.483	2.112	4.005
LobuleIX_Volume_Left	2.846	2.386	764	1467	3.596	.000	0.123	0.219	0.702
WhiteMatter_Volume_Total	51.069	45.757	842	1545	4.663	.000	0.921	3.508	7.117
Cerebellum_Volume_Right	69.926	63.580	788	1491	3.922	.000	1.397	3.607	9.084
Cerebellum_Volume_Total	139.446	127.269	786	1489	3.895	.000	2.794	6.703	17.653
GreyMatter_Volume_Total	88.377	81.511	740	1443	3.269	.001	1.985	2.976	10.756
GreyMatter_Volume_Left	44.162	40.583	745	1448	3.337	.001	1.010	1.600	5.559
GreyMatter_Volume_Right	44.215	40.928	734	1437	3.195	.001	0.989	1.348	5.225
LobuleIV_Volume_Total	2.163	2.321	273	976	−3.072	.002	0.047	−0.250	−0.067
LobuleVIIIB_Volume_Right	2.961	3.177	278	981	−3.004	.003	0.066	−0.346	−0.088
LobuleIV_Volume_Right	2.178	2.334	290	993	−2.848	.004	0.052	−0.259	−0.054
LobuleIV_Volume_Left	2.147	2.307	313	1016	−2.529	.012	0.053	−0.265	−0.055
LobuleVIIIB_Volume_Total	2.984	3.122	331	1034	−2.284	.023	0.053	−0.242	−0.033
LobuleVIIIA_Volume_Total	3.011	3.179	334	1037	−2.250	.025	0.066	−0.297	−0.038
LobuleIII_Volume_Left	1.757	1.581	662	1365	2.216	.027	0.074	0.032	0.320
LobuleIX_Volume_Total	2.758	2.467	656	1359	2.134	.033	0.118	0.061	0.522
LobuleIII_Volume_Total	1.777	1.613	653	1356	2.093	.037	0.070	0.027	0.302
LobuleVIIIA_Volume_Right	3.019	3.193	350	1053	−2.026	.043	0.073	−0.317	−0.032
LobuleVIIIA_Volume_Left	3.004	3.164	354	1057	−1.971	.049	0.068	−0.293	−0.027

This table summarizes non-parametric Mann–Whitney *U*-test results for cerebellar volumetric measures that showed significant differences between males with ASD and neurotypical male controls (ASD: n = 28; HC: n = 29). Analyses were restricted to the male subgroup defined within the fixed analytic cohort. Reported parameters include the Mann–Whitney *U* statistic (*U*), Wilcoxon rank-sum statistic (*W*), standardized *Z* value, two-tailed *P* value, standard error (SE), and 95% CI. All volumetric measures, including reported group differences and confidence intervals, were expressed in cm^3^ and normalized to TIV. Scanner- and site-related variability was mitigated using ComBat harmonization applied to the full dataset (covariates: Age, TIV). Multiple comparisons were controlled within functionally defined cerebellar lobular domains using the Benjamini–Hochberg false discovery rate (FDR; *q* < 0.05).

ASD, autism spectrum disorder; FDR, false discovery rate; HC, healthy control; SE, standard error; ciTIV, total intracranial volume; *U*, Mann–Whitney statistic; *W*, Wilcoxon rank-sum test; *Z*, standardized score.

## Data Availability

The data that support the findings of this study are available on request from the corresponding author.
