# Supplementary material for: Neuroanatomical Cerebellar Patterns in Autism Spectrum Disorder
Source: Eurasian J Med. 2026 Jun 24;58(4):e251008. doi: 10.5152/eurasianjmed.2026.251008 (PMC13338815; doi:10.5152/eurasianjmed.2026.251008)
Supplement: Supplementary Material [file supplementary_material.pdf]

Supplementary Table 1. MRI acquisition parameters across the four ABIDE sites included in this study (Caltech, CMU, KKI/HH, and Guy's Hospital).

Site	Scanner Model	Field Strength (T)	TR (ms)	TE (ms)	Flip Angle (°)	Voxel Size (mm^3)	Acquisition Type
Caltech	Siemens Trio 3T	3.0	2300	2.91	9	1.0 × 1.0 × 1.0	MPRAGE
Carnegie Mellon (CMU)	Siemens Allegra 3T	3.0	2000	3.34	8	1.1 × 1.1 × 1.1	MPRAGE
Kennedy Krieger / Johns Hopkins (KKI/HH)	Philips Achieva 3T	3.0	3500	8.5	8	1.0 × 1.0 × 1.0	T1-FFE
Guy's Hospital / Institute of Psychiatry (GUYS)	GE Signa HDx 3T	3.0	7.8	3.0	20	1.1 × 1.1 × 1.1	SPGR

This table details the structural MRI acquisition protocols for all four sites contributing to the present dataset. All scans were acquired on 3 T scanners using site-specific high-resolution T1-weighted sequences that conformed to the ABIDE consortium's minimum quality criteria, ensuring consistent anatomical coverage and adequate cerebellar contrast. Minor inter-site differences in repetition time (TR), echo time (TE), flip angle, voxel dimensions, and acquisition type were accounted for by subsequent ComBat harmonization to reduce scanner-related variance.

Abbreviations: ABIDE, Autism Brain Imaging Data Exchange; TR, Repetition Time; TE, Echo Time; SPGR, Spoiled Gradient Recall; MPRAGE, Magnetization-Prepared Rapid Gradient Echo; T1-FFE, T1-Weighted Fast Field Echo; CMU, Carnegie Mellon University; KKI/HH, Kennedy Krieger Institute/Johns Hopkins Hospital; GUYS, Guy's Hospital/Institute of Psychiatry.

Supplementary Table 2. Statistical summary of 108 cerebellar morphometric parameters before and after ComBat harmonization

Variable	p value (before)	q value (before)	Cohen's d (before)	p value (after)	q value (after)	Cohen's d (after)	Significance change	Interpretation	Clinical Effect Strength	Clinical Interpretation
LobuleIX Volume (Right)	0.088	0.152	0.343	<0.001	<0.001	0.843	Gained	Previously non-significant but became significant after ComBat correction (based on q value).	Strong	Strong clinical effect.
LobuleV Volume (Right)	<0.001	0.001	-0.773	<0.001	<0.001	-1.624	Unchanged (significant)	Remained significant before and after ComBat correction (based on q value).	Strong	Strong clinical effect.
LobuleIV Volume (Right)	<0.001	<0.001	-0.931	<0.001	<0.001	-1.903	Unchanged (significant)	Remained significant before and after ComBat correction (based on q value).	Strong	Strong clinical effect.
LobuleVIIIA Volume (Right)	0.001	0.002	-0.693	<0.001	<0.001	-1.580	Unchanged (significant)	Remained significant before and after ComBat correction (based on q value).	Strong	Strong clinical effect.
LobuleIII Thickness (Total)	0.008	0.018	0.545	<0.001	<0.001	1.131	Unchanged (significant)	Remained significant before and after ComBat correction (based on q value).	Strong	Strong clinical effect.
LobuleVIIIA Thickness (Left)	0.005	0.014	-0.570	<0.001	<0.001	-1.242	Unchanged (significant)	Remained significant before and after ComBat correction (based on q value).	Strong	Strong clinical effect.
LobuleIV Volume (Total)	<0.001	<0.001	-0.958	<0.001	<0.001	-2.044	Unchanged (significant)	Remained significant before and after ComBat correction (based on q value).	Strong	Strong clinical effect.
LobuleIV Thickness (Left)	<0.001	0.001	-0.810	<0.001	<0.001	-1.785	Unchanged (significant)	Remained significant before and after ComBat correction (based on q value).	Strong	Strong clinical effect.
LobuleIV Thickness (Total)	<0.001	<0.001	-0.958	<0.001	<0.001	-2.044	Unchanged (significant)	Remained significant before and after ComBat correction (based on q value).	Strong	Strong clinical effect.
LobuleV Thickness (Asymmetry)	<0.001	<0.001	-0.980	<0.001	<0.001	-2.030	Unchanged (significant)	Remained significant before and after ComBat correction (based on q value).	Strong	Strong clinical effect.
LobuleIII Thickness (Left)	0.007	0.017	0.560	<0.001	<0.001	1.148	Unchanged (significant)	Remained significant before and after ComBat correction (based on q value).	Strong	Strong clinical effect.
LobuleVIIIB Thickness (Right)	<0.001	0.001	-0.773	<0.001	<0.001	-1.624	Unchanged (significant)	Remained significant before and after ComBat correction (based on q value).	Strong	Strong clinical effect.
LobuleVIIIA Thickness (Right)	0.001	0.002	-0.693	<0.001	<0.001	-1.580	Unchanged (significant)	Remained significant before and after ComBat correction (based on q value).	Strong	Strong clinical effect.
LobuleIII Thickness (Left)	0.007	0.017	0.560	<0.001	<0.001	1.148	Unchanged (significant)	Remained significant before and after ComBat correction (based on q value).	Strong	Strong clinical effect.
LobuleIV Thickness (Right)	<0.001	<0.001	-0.931	<0.001	<0.001	-1.903	Unchanged (significant)	Remained significant before and after ComBat correction (based on q value).	Strong	Strong clinical effect.
LobuleVI Volume (Right)	<0.001	0.001	-0.773	<0.001	<0.001	-1.624	Unchanged (significant)	Remained significant before and after ComBat correction (based on q value).	Strong	Strong clinical effect.
LobuleVIIIA Volume (Left)	0.005	0.014	-0.570	<0.001	<0.001	-1.242	Unchanged (significant)	Remained significant before and after ComBat correction (based on q value).	Strong	Strong clinical effect.
LobuleVI Volume (Asymmetry)	<0.001	<0.001	-0.980	<0.001	<0.001	-2.030	Unchanged (significant)	Remained significant before and after ComBat correction (based on q value).	Strong	Strong clinical effect.
LobuleIV Volume (Left)	<0.001	0.001	-0.810	<0.001	<0.001	-1.785	Unchanged (significant)	Remained significant before and after ComBat correction (based on q value).	Strong	Strong clinical effect.
GreyMatter Volume (Total)	<0.001	0.001	0.741	<0.001	<0.001	1.188	Unchanged (significant)	Remained significant before and after ComBat correction (based on q value).	Strong	Strong clinical effect.

Cerebellum Volume (Total)	<0.001	<0.001	0.911	<0.001	<0.001	1.451	Unchanged (significant)	Remained significant before and after ComBat correction (based on q value).	Strong	Strong clinical effect.
LobuleVIIIA Volume (Total)	0.001	0.003	-0.666	<0.001	<0.001	-1.492	Unchanged (significant)	Remained significant before and after ComBat correction (based on q value).	Strong	Strong clinical effect.
Cerebellum Volume (Right)	<0.001	<0.001	0.947	<0.001	<0.001	1.490	Unchanged (significant)	Remained significant before and after ComBat correction (based on q value).	Strong	Strong clinical effect.
Cerebellum Volume (Left)	<0.001	<0.001	0.868	<0.001	<0.001	1.401	Unchanged (significant)	Remained significant before and after ComBat correction (based on q value).	Strong	Strong clinical effect.
Cerebellum Volume (Asymmetry)	0.005	0.013	0.582	<0.001	<0.001	1.122	Unchanged (significant)	Remained significant before and after ComBat correction (based on q value).	Strong	Strong clinical effect.
WhiteMatter Volume (Total)	<0.001	<0.001	1.191	<0.001	<0.001	1.918	Unchanged (significant)	Remained significant before and after ComBat correction (based on q value).	Strong	Strong clinical effect.
WhiteMatter Volume (Right)	<0.001	<0.001	1.307	<0.001	<0.001	2.107	Unchanged (significant)	Remained significant before and after ComBat correction (based on q value).	Strong	Strong clinical effect.
WhiteMatter Volume (Left)	<0.001	<0.001	1.028	<0.001	<0.001	1.626	Unchanged (significant)	Remained significant before and after ComBat correction (based on q value).	Strong	Strong clinical effect.
WhiteMatter Volume (Asymmetry)	<0.001	<0.001	0.870	<0.001	<0.001	1.859	Unchanged (significant)	Remained significant before and after ComBat correction (based on q value).	Strong	Strong clinical effect.
GreyMatter Volume (Right)	0.001	0.002	0.724	<0.001	<0.001	1.143	Unchanged (significant)	Remained significant before and after ComBat correction (based on q value).	Strong	Strong clinical effect.
LobuleIII Volume (Total)	0.008	0.018	0.545	<0.001	<0.001	1.131	Unchanged (significant)	Remained significant before and after ComBat correction (based on q value).	Strong	Strong clinical effect.
GreyMatter Volume (Left)	<0.001	0.001	0.752	<0.001	<0.001	1.224	Unchanged (significant)	Remained significant before and after ComBat correction (based on q value).	Strong	Strong clinical effect.
LobuleIII Volume (Left)	0.007	0.017	0.560	<0.001	<0.001	1.148	Unchanged (significant)	Remained significant before and after ComBat correction (based on q value).	Strong	Strong clinical effect.
LobuleVIIIB Thickness (Asymmetry)	<0.001	<0.001	-0.980	<0.001	<0.001	-2.030	Unchanged (significant)	Remained significant before and after ComBat correction (based on q value).	Strong	Strong clinical effect.
LobuleIII Thickness (Total)	0.008	0.018	0.545	<0.001	<0.001	1.131	Unchanged (significant)	Remained significant before and after ComBat correction (based on q value).	Strong	Strong clinical effect.
LobuleV Volume (Asymmetry)	<0.001	<0.001	-0.980	<0.001	<0.001	-2.030	Unchanged (significant)	Remained significant before and after ComBat correction (based on q value).	Strong	Strong clinical effect.
LobuleVI Thickness (Right)	<0.001	0.001	-0.773	<0.001	<0.001	-1.624	Unchanged (significant)	Remained significant before and after ComBat correction (based on q value).	Strong	Strong clinical effect.
LobuleVIIIB Volume (Total)	0.048	0.088	-0.400	<0.001	<0.001	-0.848	Gained	Previously non-significant but became significant after ComBat correction (based on q value).	Strong	Strong clinical effect.
LobuleVI Thickness (Total)	0.048	0.088	-0.400	<0.001	<0.001	-0.848	Gained	Previously non-significant but became significant after ComBat correction (based on q value).	Strong	Strong clinical effect.
LobuleIII Volume (Right)	0.025	0.054	0.456	<0.001	<0.001	0.959	Gained	Previously non-significant but became significant after ComBat correction (based on q value).	Strong	Strong clinical effect.
LobuleIII Thickness (Right)	0.025	0.054	0.456	<0.001	<0.001	0.959	Gained	Previously non-significant but became significant after ComBat correction (based on q value).	Strong	Strong clinical effect.
LobuleIII Thickness (Right)	0.025	0.054	0.456	<0.001	<0.001	0.959	Gained	Previously non-significant but became significant after ComBat correction (based on q value).	Strong	Strong clinical effect.

(*Continued*)

Supplementary Table 2. Statistical summary of 108 cerebellar morphometric parameters before and after ComBat harmonization (*Continued*)

Variable	p value (before)	q value (before)	Cohen's d (before)	p value (after)	q value (after)	Cohen's d (after)	Significance change	Interpretation	Clinical Effect Strength	Clinical Interpretation
LobuleV Thickness (Total)	0.048	0.088	-0.400	<0.001	<0.001	-0.848	Gained	Previously non-significant but became significant after ComBat correction (based on q value).	Strong	Strong clinical effect.
LobuleIX Thickness (Right)	0.088	0.152	0.343	<0.001	<0.001	0.843	Gained	Previously non-significant but became significant after ComBat correction (based on q value).	Strong	Strong clinical effect.
LobuleVIIB Thickness (Left)	0.039	0.079	-0.418	<0.001	<0.001	-0.833	Gained	Previously non-significant but became significant after ComBat correction (based on q value).	Strong	Strong clinical effect.
LobuleV Volume (Total)	0.048	0.088	-0.400	<0.001	<0.001	-0.848	Gained	Previously non-significant but became significant after ComBat correction (based on q value).	Strong	Strong clinical effect.
LobuleVIIB Volume (Left)	0.039	0.079	-0.418	<0.001	<0.001	-0.833	Gained	Previously non-significant but became significant after ComBat correction (based on q value).	Strong	Strong clinical effect.
LobuleVIIIB Thickness (Total)	0.048	0.088	-0.400	<0.001	<0.001	-0.848	Gained	Previously non-significant but became significant after ComBat correction (based on q value).	Strong	Strong clinical effect.
LobuleV Thickness (Right)	<0.001	0.001	-0.773	<0.001	<0.001	-1.624	Unchanged (significant)	Remained significant before and after ComBat correction (based on q value).	Strong	Strong clinical effect.
LobuleVIIIB Volume (Right)	<0.001	0.001	-0.773	<0.001	<0.001	-1.624	Unchanged (significant)	Remained significant before and after ComBat correction (based on q value).	Strong	Strong clinical effect.
LobuleVIIIB Volume (Asymmetry)	<0.001	<0.001	-0.980	<0.001	<0.001	-2.030	Unchanged (significant)	Remained significant before and after ComBat correction (based on q value).	Strong	Strong clinical effect.
LobuleVIIIA Thickness (Total)	0.001	0.003	-0.666	<0.001	<0.001	-1.492	Unchanged (significant)	Remained significant before and after ComBat correction (based on q value).	Strong	Strong clinical effect.
LobuleVI Thickness (Asymmetry)	<0.001	<0.001	-0.980	<0.001	<0.001	-2.030	Unchanged (significant)	Remained significant before and after ComBat correction (based on q value).	Strong	Strong clinical effect.
LobuleIX Thickness (Asymmetry)	<0.001	<0.001	-1.534	<0.001	<0.001	-3.140	Unchanged (significant)	Remained significant before and after ComBat correction (based on q value).	Strong	Strong clinical effect.
LobuleIX Thickness (Left)	<0.001	<0.001	1.027	<0.001	<0.001	2.554	Unchanged (significant)	Remained significant before and after ComBat correction (based on q value).	Strong	Strong clinical effect.
LobuleIX Volume (Total)	0.001	0.002	0.706	<0.001	<0.001	1.776	Unchanged (significant)	Remained significant before and after ComBat correction (based on q value).	Strong	Strong clinical effect.
LobuleIX Volume (Left)	<0.001	<0.001	1.027	<0.001	<0.001	2.554	Unchanged (significant)	Remained significant before and after ComBat correction (based on q value).	Strong	Strong clinical effect.
LobuleIX Volume (Asymmetry)	<0.001	<0.001	-1.534	<0.001	<0.001	-3.140	Unchanged (significant)	Remained significant before and after ComBat correction (based on q value).	Strong	Strong clinical effect.
LobuleIX Thickness (Total)	0.001	0.002	0.706	<0.001	<0.001	1.776	Unchanged (significant)	Remained significant before and after ComBat correction (based on q value).	Strong	Strong clinical effect.
LobuleVI Volume (Total)	0.048	0.088	-0.400	<0.001	<0.001	-0.848	Gained	Previously non-significant but became significant after ComBat correction (based on q value).	Strong	Strong clinical effect.
LobuleVIIB Volume (Right)	0.201	0.317	-0.256	0.008	0.012	-0.543	Gained	Previously non-significant but became significant after ComBat correction (based on q value).	Moderate	Statistically significant with moderate clinical relevance.
LobuleVIIB Volume (Total)	0.078	0.140	-0.354	<0.001	0.001	-0.731	Gained	Previously non-significant but became significant after ComBat correction (based on q value).	Moderate	Statistically significant with moderate clinical relevance.

LobuleX Volume (Total)	0.228	0.350	0.246	0.012	0.018	0.516	Gained	Previously non-significant but became significant after ComBat correction (based on q value).	Moderate	Statistically significant with moderate clinical relevance.
LobuleX Volume (Right)	0.123	0.206	0.316	0.001	0.002	0.687	Gained	Previously non-significant but became significant after ComBat correction (based on q value).	Moderate	Statistically significant with moderate clinical relevance.
LobuleVIIB Volume (Asymmetry)	0.198	0.317	0.261	0.007	0.012	0.552	Gained	Previously non-significant but became significant after ComBat correction (based on q value).	Moderate	Statistically significant with moderate clinical relevance.
LobuleVIIB Thickness (Right)	0.201	0.317	-0.256	0.008	0.012	-0.543	Gained	Previously non-significant but became significant after ComBat correction (based on q value).	Moderate	Statistically significant with moderate clinical relevance.
LobuleVIIB Thickness (Asymmetry)	0.198	0.317	0.261	0.007	0.012	0.552	Gained	Previously non-significant but became significant after ComBat correction (based on q value).	Moderate	Statistically significant with moderate clinical relevance.
LobuleX Thickness (Right)	0.123	0.206	0.316	0.001	0.002	0.687	Gained	Previously non-significant but became significant after ComBat correction (based on q value).	Moderate	Statistically significant with moderate clinical relevance.
LobuleVIIB Thickness (Total)	0.078	0.140	-0.354	<0.001	0.001	-0.731	Gained	Previously non-significant but became significant after ComBat correction (based on q value).	Moderate	Statistically significant with moderate clinical relevance.
LobuleX Thickness (Total)	0.228	0.350	0.246	0.012	0.018	0.516	Gained	Previously non-significant but became significant after ComBat correction (based on q value).	Moderate	Statistically significant with moderate clinical relevance.
LobuleVIIIA Thickness (Asymmetry)	0.284	0.425	-0.218	0.029	0.043	-0.445	Gained	Previously non-significant but became significant after ComBat correction (based on q value).	Small	Statistically significant but clinically weak.
LobuleVIIIA Volume (Asymmetry)	0.284	0.425	-0.218	0.029	0.043	-0.445	Gained	Previously non-significant but became significant after ComBat correction (based on q value).	Small	Statistically significant but clinically weak.
CrusI Volume (Asymmetry)	0.855	0.879	-0.037	0.843	0.843	-0.040	Unchanged (non-significant)	Non-significant both before and after ComBat correction (based on q value).	Negligible	Not statistically significant.
LobuleX Volume (Left)	0.533	0.652	0.126	0.215	0.263	0.251	Unchanged (non-significant)	Non-significant both before and after ComBat correction (based on q value).	Small	Not statistically significant.
LobuleX Volume (Asymmetry)	0.374	0.493	0.177	0.050	0.073	0.396	Unchanged (non-significant)	Non-significant both before and after ComBat correction (based on q value).	Small	Not statistically significant.
CrusI Volume (Left)	0.421	0.528	-0.160	0.123	0.155	-0.310	Unchanged (non-significant)	Non-significant both before and after ComBat correction (based on q value).	Small	Not statistically significant.
CrusI Volume (Right)	0.369	0.493	-0.179	0.077	0.107	-0.356	Unchanged (non-significant)	Non-significant both before and after ComBat correction (based on q value).	Small	Not statistically significant.
CrusI Volume (Total)	0.375	0.493	-0.177	0.084	0.111	-0.348	Unchanged (non-significant)	Non-significant both before and after ComBat correction (based on q value).	Small	Not statistically significant.
LobuleIII Thickness (Asymmetry)	0.920	0.920	-0.020	0.787	0.817	-0.054	Unchanged (non-significant)	Non-significant both before and after ComBat correction (based on q value).	Negligible	Not statistically significant.
LobuleV Volume (Left)	0.649	0.737	0.091	0.324	0.372	0.198	Unchanged (non-significant)	Non-significant both before and after ComBat correction (based on q value).	Negligible	Not statistically significant.
LobuleIII Thickness (Asymmetry)	0.920	0.920	-0.020	0.787	0.817	-0.054	Unchanged (non-significant)	Non-significant both before and after ComBat correction (based on q value).	Negligible	Not statistically significant.

(*Continued*)

Supplementary Table 2. Statistical summary of 108 cerebellar morphometric parameters before and after ComBat harmonization (*Continued*)

Variable	p value (before)	q value (before)	Cohen's d (before)	p value (after)	q value (after)	Cohen's d (after)	Significance change	Interpretation	Clinical Effect Strength	Clinical Interpretation
CrusII Volume (Total)	0.375	0.493	-0.177	0.084	0.111	-0.348	Unchanged (non-significant)	Non-significant both before and after ComBat correction (based on q value).	Small	Not statistically significant.
LobuleIV Thickness (Asymmetry)	0.821	0.879	-0.046	0.725	0.775	-0.071	Unchanged (non-significant)	Non-significant both before and after ComBat correction (based on q value).	Negligible	Not statistically significant.
LobuleIV Volume (Asymmetry)	0.821	0.879	-0.046	0.725	0.775	-0.071	Unchanged (non-significant)	Non-significant both before and after ComBat correction (based on q value).	Negligible	Not statistically significant.
LobuleV Thickness (Left)	0.649	0.737	0.091	0.324	0.372	0.198	Unchanged (non-significant)	Non-significant both before and after ComBat correction (based on q value).	Negligible	Not statistically significant.
LobuleIII Volume (Asymmetry)	0.920	0.920	-0.020	0.787	0.817	-0.054	Unchanged (non-significant)	Non-significant both before and after ComBat correction (based on q value).	Negligible	Not statistically significant.
CrusI Thickness (Total)	0.375	0.493	-0.177	0.084	0.111	-0.348	Unchanged (non-significant)	Non-significant both before and after ComBat correction (based on q value).	Small	Not statistically significant.
LobuleVI Volume (Left)	0.649	0.737	0.091	0.324	0.372	0.198	Unchanged (non-significant)	Non-significant both before and after ComBat correction (based on q value).	Negligible	Not statistically significant.
CrusII Volume (Right)	0.369	0.493	-0.179	0.077	0.107	-0.356	Unchanged (non-significant)	Non-significant both before and after ComBat correction (based on q value).	Small	Not statistically significant.
CrusII Volume (Asymmetry)	0.855	0.879	-0.037	0.843	0.843	-0.040	Unchanged (non-significant)	Non-significant both before and after ComBat correction (based on q value).	Negligible	Not statistically significant.
LobuleX Thickness (Left)	0.533	0.652	0.126	0.215	0.263	0.251	Unchanged (non-significant)	Non-significant both before and after ComBat correction (based on q value).	Small	Not statistically significant.
CrusI Thickness (Left)	0.421	0.528	-0.160	0.123	0.155	-0.310	Unchanged (non-significant)	Non-significant both before and after ComBat correction (based on q value).	Small	Not statistically significant.
CrusII Thickness (Right)	0.369	0.493	-0.179	0.077	0.107	-0.356	Unchanged (non-significant)	Non-significant both before and after ComBat correction (based on q value).	Small	Not statistically significant.
CrusII Thickness (Left)	0.421	0.528	-0.160	0.123	0.155	-0.310	Unchanged (non-significant)	Non-significant both before and after ComBat correction (based on q value).	Small	Not statistically significant.
LobuleIII Volume (Total)	0.775	0.862	-0.058	0.552	0.614	-0.120	Unchanged (non-significant)	Non-significant both before and after ComBat correction (based on q value).	Negligible	Not statistically significant.
LobuleIII Volume (Asymmetry)	0.838	0.879	-0.042	0.672	0.732	-0.086	Unchanged (non-significant)	Non-significant both before and after ComBat correction (based on q value).	Negligible	Not statistically significant.
LobuleIII Volume (Left)	0.828	0.879	-0.044	0.652	0.717	-0.091	Unchanged (non-significant)	Non-significant both before and after ComBat correction (based on q value).	Negligible	Not statistically significant.
LobuleIII Volume (Right)	0.719	0.808	-0.073	0.455	0.511	-0.151	Unchanged (non-significant)	Non-significant both before and after ComBat correction (based on q value).	Negligible	Not statistically significant.
CrusII Thickness (Total)	0.375	0.493	-0.177	0.084	0.111	-0.348	Unchanged (non-significant)	Non-significant both before and after ComBat correction (based on q value).	Small	Not statistically significant.
GreyMatter Volume (Asymmetry)	0.613	0.737	-0.102	0.365	0.414	-0.182	Unchanged (non-significant)	Non-significant both before and after ComBat correction (based on q value).	Negligible	Not statistically significant.
LobuleVIIIB Thickness (Left)	0.649	0.737	0.091	0.324	0.372	0.198	Unchanged (non-significant)	Non-significant both before and after ComBat correction (based on q value).	Negligible	Not statistically significant.
CrusII Thickness (Asymmetry)	0.855	0.879	-0.037	0.843	0.843	-0.040	Unchanged (non-significant)	Non-significant both before and after ComBat correction (based on q value).	Negligible	Not statistically significant.
CrusI Thickness (Asymmetry)	0.855	0.879	-0.037	0.843	0.843	-0.040	Unchanged (non-significant)	Non-significant both before and after ComBat correction (based on q value).	Negligible	Not statistically significant.

LobuleVI Thickness (Left)	0.649	0.737	0.091	0.324	0.372	0.198	Unchanged (non-significant)	Non-significant both before and after ComBat correction (based on q value).	Negligible	Not statistically significant.
CrusI Thickness (Right)	0.369	0.493	-0.179	0.077	0.107	-0.356	Unchanged (non-significant)	Non-significant both before and after ComBat correction (based on q value).	Small	Not statistically significant.
LobuleVIIIB Volume (Left)	0.649	0.737	0.091	0.324	0.372	0.198	Unchanged (non-significant)	Non-significant both before and after ComBat correction (based on q value).	Negligible	Not statistically significant.
CrusII Volume (Left)	0.421	0.528	-0.160	0.123	0.155	-0.310	Unchanged (non-significant)	Non-significant both before and after ComBat correction (based on q value).	Small	Not statistically significant.
LobuleX Thickness (Asymmetry)	0.374	0.493	0.177	0.050	0.073	0.396	Unchanged (non-significant)	Non-significant both before and after ComBat correction (based on q value).	Small	Not statistically significant.

This table summarizes statistical and clinical outcomes for 108 cerebellar morphometric parameters analyzed before and after ComBat harmonization. Each variable includes unharmonized and harmonized p-values, FDR-adjusted q-values, and corresponding effect sizes (Cohen's d). FDR decisions (q-values) refer to tests run on the ComBat-harmonized dataset, unless otherwise noted. The Significance change column indicates whether a variable gained, lost, or retained statistical significance after harmonization, while the Interpretation and Clinical Interpretation columns provide concise explanations of the statistical and clinical meaning of each result.
ComBat harmonization was performed using an empirical Bayes approach to remove scanner- and site-related variance while preserving biologically relevant effects associated with diagnosis, sex, age, and total intracranial volume (TIV). Among the 108 variables examined, 14 newly reached statistical significance following harmonization, confirming that variance correction improved detection of subtle yet biologically meaningful differences. Importantly, no parameter reversed its direction of effect, demonstrating that the harmonization process preserved true neuroanatomical patterns rather than introducing artifacts.
Clinically, large or moderate effect sizes ($d \geq 0.8$) correspond to robust cerebellar alterations—most prominently in Lobules IV–V, VIIIA–B, IX, and cerebellar white matter—supporting their potential as reproducible neuroanatomical markers of ASD. Effect size thresholds; $|d| \geq 0.8$ = Strong; 0.5–0.79 = Moderate; 0.2–0.49 = Small; <0.2 = Negligible. Statistical significance set at $q < 0.05$ after FDR correction.
No direction reversals were observed: Variables that were positive or negative before ComBat retained their sign after harmonization, confirming stability of the biological effect direction.
Abbreviations: ASD, Autism Spectrum Disorder; HC, Healthy Control; FDR, False Discovery Rate; d, Cohen's d (effect size); q, FDR-adjusted p-value; TIV, Total Intracranial Volume; ComBat, Combined Batch Effect Adjustment.

Supplementary Table 3. Non-significant parameters after FDR (q ≥ 0.05) on the ComBat-harmonized dataset

Variable	Group	N	Mean	SD	Min	Max
Cerebellum Volume(Asymmetry)	ASD	48	0.448	1.975	-4.536	5.222
Cerebellum Volume(Asymmetry)	HC	52	-0.627	1.717	-3.486	3.738
Crus II Thickness(Asymmetry)	ASD	48	0.053	4.811	-9.313	19.913
Crus II Thickness(Asymmetry)	HC	52	0.230	4.827	-13.054	9.809
Crus II Thickness(Left)	ASD	48	3.160	0.224	2.670	3.720
Crus II Thickness(Left)	HC	52	3.200	0.271	2.690	3.800
Crus II Thickness(Right)	ASD	48	3.162	0.229	2.680	3.890
Crus II Thickness(Right)	HC	52	3.210	0.298	2.540	3.750
Crus II Thickness(Total)	ASD	48	3.161	0.213	2.690	3.750
Crus II Thickness(Total)	HC	52	3.205	0.276	2.720	3.720
Crus II Volume(Asymmetry)	ASD	48	0.053	4.811	-9.313	19.913
Crus II Volume(Asymmetry)	HC	52	0.230	4.827	-13.054	9.809
Crus II Volume(Left)	ASD	48	3.160	0.224	2.670	3.720
Crus II Volume(Left)	HC	52	3.200	0.271	2.690	3.800
Crus II Volume(Right)	ASD	48	3.162	0.229	2.680	3.890
Crus II Volume(Right)	HC	52	3.210	0.298	2.540	3.750
Crus II Volume(Total)	ASD	48	3.161	0.213	2.690	3.750
Crus II Volume(Total)	HC	52	3.205	0.276	2.720	3.720
Crus I Thickness(Asymmetry)	ASD	48	0.053	4.811	-9.313	19.913
Crus I Thickness(Asymmetry)	HC	52	0.230	4.827	-13.054	9.809
Crus I Thickness(Left)	ASD	48	3.160	0.224	2.670	3.720
Crus I Thickness(Left)	HC	52	3.200	0.271	2.690	3.800
Crus I Thickness(Right)	ASD	48	3.162	0.229	2.680	3.890
Crus I Thickness(Right)	HC	52	3.210	0.298	2.540	3.750
Crus I Thickness(Total)	ASD	48	3.161	0.213	2.690	3.750
Crus I Thickness(Total)	HC	52	3.205	0.276	2.720	3.720
Crus I Volume(Asymmetry)	ASD	48	0.053	4.811	-9.313	19.913
Crus I Volume(Asymmetry)	HC	52	0.230	4.827	-13.054	9.809
Crus I Volume(Left)	ASD	48	3.160	0.224	2.670	3.720
Crus I Volume(Left)	HC	52	3.200	0.271	2.690	3.800
Crus I Volume(Right)	ASD	48	3.162	0.229	2.680	3.890
Crus I Volume(Right)	HC	52	3.210	0.298	2.540	3.750
Crus I Volume(Total)	ASD	48	3.161	0.213	2.690	3.750
Crus I Volume(Total)	HC	52	3.205	0.276	2.720	3.720
Cerebellar Gray Matter Volume(Asymmetry)	ASD	48	0.032	2.136	-5.664	6.998
Cerebellar Gray Matter Volume(Asymmetry)	HC	52	0.232	1.773	-3.235	4.034
Lobule III Thickness(Asymmetry)	ASD	48	2.411	14.657	-18.350	53.396
Lobule III Thickness(Asymmetry)	HC	52	2.702	14.000	-51.716	33.954
Lobule III Thickness(Left)	ASD	48	1.763	0.347	0.740	2.840
Lobule III Thickness(Left)	HC	52	1.592	0.263	0.960	2.170
Lobule III Thickness(Right)	ASD	48	1.804	0.354	1.080	2.880
Lobule III Thickness(Right)	HC	52	1.649	0.326	0.580	2.330
Lobule III Thickness(Total)	ASD	48	1.785	0.331	1.010	2.860

Lobule III Thickness(Total)	HC	52	1.619	0.278	0.780	2.210
Lobule III Volume(Asymmetry)	ASD	48	2.411	14.657	-18.350	53.396
Lobule III Volume(Asymmetry)	HC	52	2.702	14.000	-51.716	33.954
Lobule III Volume(Left)	ASD	48	1.763	0.347	0.740	2.840
Lobule III Volume(Left)	HC	52	1.592	0.263	0.960	2.170
Lobule III Volume(Right)	ASD	48	1.804	0.354	1.080	2.880
Lobule III Volume(Right)	HC	52	1.649	0.326	0.580	2.330
Lobule III Volume(Total)	ASD	48	1.785	0.331	1.010	2.860
Lobule III Volume(Total)	HC	52	1.619	0.278	0.780	2.210
Lobule IV Thickness(Asymmetry)	ASD	48	1.222	10.122	-19.116	22.614
Lobule IV Thickness(Asymmetry)	HC	52	1.613	6.620	-17.887	19.144
Lobule IV Thickness(Left)	ASD	48	2.156	0.257	1.560	2.630
Lobule IV Thickness(Left)	HC	52	2.336	0.185	1.880	2.790
Lobule IV Thickness(Right)	ASD	48	2.178	0.233	1.660	2.710
Lobule IV Thickness(Right)	HC	52	2.374	0.187	1.960	2.670
Lobule IV Thickness(Total)	ASD	48	2.167	0.220	1.720	2.570
Lobule IV Thickness(Total)	HC	52	2.355	0.171	1.920	2.730
Lobule IV Volume(Asymmetry)	ASD	48	1.222	10.122	-19.116	22.614
Lobule IV Volume(Asymmetry)	HC	52	1.613	6.620	-17.887	19.144
Lobule IV Volume(Left)	ASD	48	2.156	0.257	1.560	2.630
Lobule IV Volume(Left)	HC	52	2.336	0.185	1.880	2.790
Lobule IV Volume(Right)	ASD	48	2.178	0.233	1.660	2.710
Lobule IV Volume(Right)	HC	52	2.374	0.187	1.960	2.670
Lobule IV Volume(Total)	ASD	48	2.167	0.220	1.720	2.570
Lobule IV Volume(Total)	HC	52	2.355	0.171	1.920	2.730
Lobule IX Thickness(Right)	ASD	48	2.691	0.411	1.680	3.240
Lobule IX Thickness(Right)	HC	52	2.536	0.486	1.540	3.390
Lobule IX Volume(Right)	ASD	48	2.691	0.411	1.680	3.240
Lobule IX Volume(Right)	HC	52	2.536	0.486	1.540	3.390
Lobule VIIB Thickness(Asymmetry)	ASD	48	1.497	6.129	-13.311	18.741
Lobule VIIB Thickness(Asymmetry)	HC	52	0.011	5.268	-9.513	9.844
Lobule VIIB Thickness(Left)	ASD	48	3.194	0.284	2.530	4.080
Lobule VIIB Thickness(Left)	HC	52	3.319	0.312	2.750	4.060
Lobule VIIB Thickness(Right)	ASD	48	3.242	0.284	2.620	4.310
Lobule VIIB Thickness(Right)	HC	52	3.322	0.339	2.600	4.120
Lobule VIIB Thickness(Total)	ASD	48	3.218	0.267	2.640	4.190
Lobule VIIB Thickness(Total)	HC	52	3.321	0.314	2.670	4.090
Lobule VIIB Volume(Asymmetry)	ASD	48	1.497	6.129	-13.311	18.741
Lobule VIIB Volume(Asymmetry)	HC	52	0.011	5.268	-9.513	9.844
Lobule VIIB Volume(Left)	ASD	48	3.194	0.284	2.530	4.080
Lobule VIIB Volume(Left)	HC	52	3.319	0.312	2.750	4.060
Lobule VIIB Volume(Right)	ASD	48	3.242	0.284	2.620	4.310
Lobule VIIB Volume(Right)	HC	52	3.322	0.339	2.600	4.120
Lobule VIIB Volume(Total)	ASD	48	3.218	0.267	2.640	4.190
Lobule VIIB Volume(Total)	HC	52	3.321	0.314	2.670	4.090

Lobule VIIIA Thickness(Asymmetry)	ASD	48	-0.016	6.633	-17.729	15.242
Lobule VIIIA Thickness(Asymmetry)	HC	52	1.260	5.025	-11.172	14.002
Lobule VIIIA Thickness(Left)	ASD	48	3.016	0.291	2.330	3.730
Lobule VIIIA Thickness(Left)	HC	52	3.185	0.300	2.360	3.730
Lobule VIIIA Thickness(Right)	ASD	48	3.015	0.294	2.410	3.820
Lobule VIIIA Thickness(Right)	HC	52	3.226	0.314	2.540	3.870
Lobule VIIIA Thickness(Total)	ASD	48	3.015	0.276	2.490	3.770
Lobule VIIIA Thickness(Total)	HC	52	3.206	0.296	2.500	3.800
Lobule VIIIA Volume(Asymmetry)	ASD	48	-0.016	6.633	-17.729	15.242
Lobule VIIIA Volume(Asymmetry)	HC	52	1.260	5.025	-11.172	14.002
Lobule VIIIA Volume(Left)	ASD	48	3.016	0.291	2.330	3.730
Lobule VIIIA Volume(Left)	HC	52	3.185	0.300	2.360	3.730
Lobule VIIIA Volume(Right)	ASD	48	3.015	0.294	2.410	3.820
Lobule VIIIA Volume(Right)	HC	52	3.226	0.314	2.540	3.870
Lobule VIIIA Volume(Total)	ASD	48	3.015	0.276	2.490	3.770
Lobule VIIIA Volume(Total)	HC	52	3.206	0.296	2.500	3.800
Lobule VIIIB Thickness(Left)	ASD	48	3.043	0.232	2.510	3.550
Lobule VIIIB Thickness(Left)	HC	52	3.020	0.269	2.170	3.480
Lobule VIIIB Thickness(Total)	ASD	48	3.013	0.222	2.530	3.560
Lobule VIIIB Thickness(Total)	HC	52	3.107	0.248	2.270	3.600
Lobule VIIIB Volume(Left)	ASD	48	3.043	0.232	2.510	3.550
Lobule VIIIB Volume(Left)	HC	52	3.020	0.269	2.170	3.480
Lobule VIIIB Volume(Total)	ASD	48	3.013	0.222	2.530	3.560
Lobule VIIIB Volume(Total)	HC	52	3.107	0.248	2.270	3.600
Lobule VI Thickness(Left)	ASD	48	3.043	0.232	2.510	3.550
Lobule VI Thickness(Left)	HC	52	3.020	0.269	2.170	3.480
Lobule VI Thickness(Total)	ASD	48	3.013	0.222	2.530	3.560
Lobule VI Thickness(Total)	HC	52	3.107	0.248	2.270	3.600
Lobule VI Volume(Left)	ASD	48	3.043	0.232	2.510	3.550
Lobule VI Volume(Left)	HC	52	3.020	0.269	2.170	3.480
Lobule VI Volume(Total)	ASD	48	3.013	0.222	2.530	3.560
Lobule VI Volume(Total)	HC	52	3.107	0.248	2.270	3.600
Lobule V Thickness(Left)	ASD	48	3.043	0.232	2.510	3.550
Lobule V Thickness(Left)	HC	52	3.020	0.269	2.170	3.480
Lobule V Thickness(Total)	ASD	48	3.013	0.222	2.530	3.560
Lobule V Thickness(Total)	HC	52	3.107	0.248	2.270	3.600
Lobule V Volume(Left)	ASD	48	3.043	0.232	2.510	3.550
Lobule V Volume(Left)	HC	52	3.020	0.269	2.170	3.480
Lobule V Volume(Total)	ASD	48	3.013	0.222	2.530	3.560
Lobule V Volume(Total)	HC	52	3.107	0.248	2.270	3.600
Lobule X Thickness(Asymmetry)	ASD	48	0.915	7.306	-15.988	17.639
Lobule X Thickness(Asymmetry)	HC	52	-0.524	8.807	-17.716	24.409
Lobule X Thickness(Left)	ASD	48	3.699	0.350	2.820	4.570
Lobule X Thickness(Left)	HC	52	3.658	0.306	2.700	4.100

Lobule X Thickness(Right)	ASD	48	3.734	0.359	2.740	4.350
Lobule X Thickness(Right)	HC	52	3.635	0.263	3.090	4.120
Lobule X Thickness(Total)	ASD	48	3.716	0.329	2.980	4.460
Lobule X Thickness(Total)	HC	52	3.646	0.238	3.040	4.080
Lobule X Volume(Asymmetry)	ASD	48	0.915	7.306	-15.988	17.639
Lobule X Volume(Asymmetry)	HC	52	-0.524	8.807	-17.716	24.409
Lobule X Volume(Left)	ASD	48	3.699	0.350	2.820	4.570
Lobule X Volume(Left)	HC	52	3.658	0.306	2.700	4.100
Lobule X Volume(Right)	ASD	48	3.734	0.359	2.740	4.350
Lobule X Volume(Right)	HC	52	3.635	0.263	3.090	4.120
Lobule X Volume(Total)	ASD	48	3.716	0.329	2.980	4.460
Lobule X Volume(Total)	HC	52	3.646	0.238	3.040	4.080
Lobule III Thickness(Asymmetry)	ASD	48	2.411	14.657	-18.350	53.396
Lobule III Thickness(Asymmetry)	HC	52	2.702	14.000	-51.716	33.954
Lobule III Thickness(Left)	ASD	48	1.763	0.347	0.740	2.840
Lobule III Thickness(Left)	HC	52	1.592	0.263	0.960	2.170
Lobule III Thickness(Right)	ASD	48	1.804	0.354	1.080	2.880
Lobule III Thickness(Right)	HC	52	1.649	0.326	0.580	2.330
Lobule III Thickness(Total)	ASD	48	1.785	0.331	1.010	2.860
Lobule III Thickness(Total)	HC	52	1.619	0.278	0.780	2.210
Lobule III Volume(Asymmetry)	ASD	48	6.656	18.240	-29.951	61.671
Lobule III Volume(Asymmetry)	HC	52	7.301	12.423	-27.829	46.219
Lobule III Volume(Left)	ASD	48	1.660	0.945	0.280	4.050
Lobule III Volume(Left)	HC	52	1.698	0.772	0.450	4.100
Lobule III Volume(Right)	ASD	48	1.737	0.950	0.420	3.970
Lobule III Volume(Right)	HC	52	1.799	0.747	0.520	4.160
Lobule III Volume(Total)	ASD	48	1.699	0.943	0.400	4.010
Lobule III Volume(Total)	HC	52	1.748	0.754	0.480	4.130
Total Intracranial Volume (TIV)	ASD	48	1527.891	169.312	1087.250	1923.860
Total Intracranial Volume (TIV)	HC	52	1399.629	137.487	1151.050	1698.300
Cerebellar White Matter Volume(Left)	ASD	48	24.608	2.359	18.630	28.900
Cerebellar White Matter Volume(Left)	HC	52	22.211	2.307	16.310	27.150
Cerebellar White Matter Volume(Right)	ASD	48	24.928	2.630	17.210	29.900
Cerebellar White Matter Volume(Right)	HC	52	21.729	2.266	15.970	26.230
Cerebellar White Matter Volume(Total)	ASD	48	49.536	4.916	36.080	58.800
Cerebellar White Matter Volume(Total)	HC	52	43.940	4.486	32.280	53.380

This table lists the cerebellar morphometric parameters that did not show statistically significant group differences between adults with ASD and healthy controls after ComBat harmonization and FDR correction. For each variable, group-wise descriptive statistics (mean ± SD, minimum, maximum) are provided to ensure full transparency of the dataset and facilitate comparison with previously published findings. These non-significant results include volumetric, cortical-thickness, and asymmetry measures across multiple cerebellar lobules (e.g., Crus I–II, Lobules III–X, and white-matter subregions).
Although these parameters did not survive multiple-comparison correction, several exhibited numerically consistent effect directions favoring mild volumetric or thickness increases in ASD. Reporting them here maintains methodological completeness and supports meta-analytic reproducibility.
Abbreviations: ASD, Autism Spectrum Disorder; HC, Healthy Control; SD, Standard Deviation; Min, Minimum value; Max, Maximum value; TIV, Total Intracranial Volume.

Supplementary Table 4. Independent-samples *t*-test results for cerebellar pseudo-cortical thickness measures and volumetric asymmetry indices showing significant between-group differences after ComBat harmonization.

Variable	F	t	Cohen's d	Interpretation	df	p	MD	SE	CI Lower	CI Upper
LobuleIV_Thickness_Total	3.483	-4.786	-0.97-0.97	Largeeffect	98	0.000	-0.188	0.039	-0.266	-0.110
LobuleIV_Thickness_Right	1.875	-4.652	-0.94-0.94	Largeeffect	98	0.000	-0.196	0.042	-0.279	-0.112
LobuleIV_Thickness_Left	5.891	-4.046	-0.82-0.82	Largeeffect	98	0.000	-0.180	0.045	-0.268	-0.092
LobuleVIIIA_Thickness_Right	0.541	-3.462	-0.70-0.70	Mediumeffect	98	0.001	-0.211	0.061	-0.332	-0.090
LobuleVIIIA_Thickness_Total	0.596	-3.327	-0.67-0.67	Mediumeffect	98	0.001	-0.191	0.057	-0.305	-0.077
Cerebellum_Volume_Asymmetry	0.021	2.909	0.590.59	Mediumeffect	98	0.004	1.074	0.369	0.341	1.807
LobuleVIIIA_Thickness_Left	0.145	-2.850	-0.58-0.58	Mediumeffect	98	0.005	-0.169	0.059	-0.286	-0.051
LobuleIII_Thickness_Left	3.008	2.800	0.570.57	Mediumeffect	98	0.006	0.172	0.061	0.050	0.294
LobuleIII_Thickness_Total	1.471	2.724	0.550.55	Mediumeffect	98	0.008	0.166	0.061	0.045	0.287
LobuleIII_Thickness_Right	0.834	2.278	0.460.46	Smalleffect	98	0.025	0.155	0.068	0.020	0.290
LobuleVIIB_Thickness_Left	1.283	-2.089	-0.42-0.42	Smalleffect	98	0.039	-0.125	0.060	-0.243	-0.006
LobuleV_Thickness_Total	0.334	-1.996	-0.40-0.40	Smalleffect	98	0.049	-0.094	0.047	-0.188	-0.001

This table presents parametric independent-samples *t*-test results for cerebellar pseudo-cortical thickness measures and volumetric asymmetry indices that showed significant differences between adults with autism spectrum disorder (ASD) and neurotypical controls. Reported statistics include mean ± SD, t value, degrees of freedom (df), p value, and Cohen's d as an effect-size indicator. F values correspond to Levene's tests for equality of variances. Pseudo-cortical thickness measures were normalized using the cubic root of TIV, whereas asymmetry indices were computed from ComBat-harmonized hemispheric measures and are inherently scale-independent.

Scanner- and site-related variability was mitigated using ComBat harmonization was applied to the full dataset prior to group comparisons, with sex, age, and TIV included as covariates. Multiple comparisons were controlled within functionally defined cerebellar lobular domains using the Benjamini–Hochberg false discovery rate (FDR; $q < 0.05$).

Abbreviations: ASD, Autism Spectrum Disorder; HC, Healthy Control; TIV, Total Intracranial Volume; SD, Standard Deviation; SE, Standard Error; CI, Confidence Interval; FDR, False Discovery Rate.

Supplementary Table 5. Sex-stratified group comparisons of cerebellar volumetric measures, volume-derived asymmetry indices, and pseudo-cortical thickness measures between females with autism spectrum disorder (ASD) and healthy controls using Mann–Whitney U tests ($p < 0.05$).

Variable	ASD Mean	HC Mean	U	W	Z	p	SE	CI Lower	CI Upper
LobuleVI_Volume_Asymmetry	-3.407	7.708	32	98	-3.623	0.000	2.529	-16.072	-6.159
LobuleIX_Volume_Asymmetry	-7.319	5.321	38	104	-3.417	0.001	3.179	-18.871	-6.409
LobuleIX_Volume_Left	2.964	2.406	223	289	2.953	0.003	0.138	0.287	0.829
WhiteMatter_Volume_Asymmetry	0.114	-0.441	207	273	2.387	0.018	0.287	-0.007	1.118
LobuleIX_Thickness_Total	2.864	2.466	205	271	2.318	0.021	0.138	0.127	0.668
LobuleIV_Thickness_Total	2.182	2.392	73	139	-2.215	0.028	0.089	-0.384	-0.036
LobuleVIIIA_Volume_Asymmetry	-1.874	1.796	75	141	-2.146	0.033	1.602	-6.810	-0.530

This supplementary table presents non-parametric Mann–Whitney U test results for cerebellar volumetric measures, volume-derived asymmetry indices, and pseudo-cortical thickness measures that showed significant differences between females with autism spectrum disorder (ASD) and neurotypical female controls. Analyses were restricted to the female-subgroup defined within the fixed analytic cohort.
Reported statistics include the Mann–Whitney U statistic (U), Wilcoxon rank-sum statistic (W), standardized Z value, two-tailed p value, standard error (SE), and 95% confidence intervals (CI). Volumetric measures were normalized to total intracranial volume (TIV; $mm^3 \times 10^3$), pseudo-cortical thickness measures were normalized using the cubic root of TIV, and asymmetry indices were computed from ComBat-harmonized hemispheric measures and are inherently scale-independent.
To control for scanner- and site-related variability, ComBat harmonization was performed on the full cohort prior to sex-stratified analyses, with age and TIV included as covariates. Multiple comparisons were corrected within functionally defined cerebellar lobular domains using the Benjamini–Hochberg false discovery rate (FDR; $q < 0.05$).
Abbreviations: ASD, Autism Spectrum Disorder; HC, Healthy Control; U, Mann–Whitney statistic; W, Wilcoxon rank-sum statistic; Z, standardized score; SE, standard error; CI, confidence interval; TIV, total intracranial volume; FDR, false discovery rate.

Supplementary Table 6. Sex-stratified group comparisons of cerebellar pseudo-cortical thickness measures and volume-based asymmetry indices between males with ASD and healthy controls using Mann–Whitney U tests ($p < 0.05$).

Variable	ASD Mean	HC Mean	U	W	Z	p	SE	CI Lower	CI Upper
LobuleIX_Volume_Asymmetry	-6.647	7.236	100	803	-5.431	0.000	2.099	-17.997	-9.769
LobuleIX_Thickness_Left	2.846	2.386	764	1467	3.596	0.000	0.123	0.219	0.702
LobuleIV_Thickness_Total	2.163	2.321	273	976	-3.072	0.002	0.047	-0.250	-0.067
LobuleVIIIB_Thickness_Right	2.961	3.177	278	981	-3.004	0.003	0.066	-0.346	-0.088
WhiteMatter_Volume_Asymmetry	0.241	-0.349	709	1412	2.848	0.004	0.190	0.217	0.963
LobuleIV_Thickness_Right	2.178	2.334	290	993	-2.848	0.004	0.052	-0.259	-0.054
LobuleIV_Thickness_Left	2.147	2.307	313	1016	-2.529	0.012	0.053	-0.265	-0.055
LobuleV_Thickness_Total	2.984	3.122	331	1034	-2.284	0.023	0.053	-0.242	-0.033
LobulesI-II_Thickness_Left	1.757	1.581	662	1365	2.216	0.027	0.074	0.032	0.320
LobuleIX_Thickness_Total	2.758	2.467	656	1359	2.134	0.033	0.118	0.061	0.522
LobuleVIIIB_Volume_Asymmetry	-1.788	3.649	343	1046	-2.127	0.034	2.007	-9.370	-1.504
LobuleIII_Thickness_Total	1.777	1.613	653	1356	2.093	0.037	0.070	0.027	0.302
LobuleVIIIA_Thickness_Right	3.019	3.193	350	1053	-2.026	0.043	0.073	-0.317	-0.032
LobuleVIIIA_Thickness_Left	3.004	3.164	354	1057	-1.971	0.049	0.068	-0.293	-0.027

This supplementary table presents non-parametric Mann–Whitney U test results for cerebellar pseudo-cortical thickness measures and volume-based asymmetry indices that showed significant differences between males with ASD and neurotypical male controls. Analyses were restricted to the male subgroup defined within the fixed analytic cohort.
Reported statistics include the Mann–Whitney U statistic (U), Wilcoxon rank-sum statistic (W), standardized Z value, two-tailed p value, standard error (SE), and 95% confidence intervals (CI). Pseudo-cortical thickness measures were normalized using the cubic root of TIV, whereas volumetric asymmetry indices were computed from TIV-normalized volumes.
Scanner- and site-related variability was mitigated using ComBat harmonization performed on the full cohort prior to sex-stratified analyses, with age and TIV included as covariates. Multiple comparisons were controlled within functionally defined cerebellar lobular domains using the Benjamini–Hochberg false discovery rate (FDR; $q < 0.05$).
Abbreviations: ASD, Autism Spectrum Disorder; HC, Healthy Control; U, Mann–Whitney statistic; W, Wilcoxon rank-sum statistic; Z, standardized score; SE, standard error; CI, confidence interval; TIV, total intracranial volume; FDR, false discovery rate.
